# Oligomerization and positive feedback on membrane recruitment encode dynamically stable PAR-3 asymmetries in the *C. elegans* zygote

**DOI:** 10.1101/2023.08.04.552031

**Published:** 2024-08-28

**Authors:** Charlie Lang, Ondrej Maxian, Alexander Anneken, Edwin Munro

**Affiliations:** 1 Department of Molecular Genetics and Cell Biology, University of Chicago, Chicago, IL 60637; 2 Committee on Genetics, Genomics and Systems Biology, University of Chicago, Chicago, IL 60637; 3 Institute for Biophysical Dynamics, University of Chicago, Chicago, IL 60637; 4 Current address: Department of Molecular and Cellular Physiology, Stanford University, Stanford, CA 94305

## Abstract

Studies of PAR polarity have emphasized a paradigm in which mutually antagonistic PAR proteins form complementary polar domains in response to transient cues. A growing body of work suggests that the oligomeric scaffold PAR-3 can form unipolar asymmetries without mutual antagonism, but how it does so is largely unknown. Here we combine single molecule analysis and modeling to show how the interplay of two positive feedback loops promote dynamically stable unipolar PAR-3 asymmetries in early *C. elegans* embryos. First, the intrinsic dynamics of PAR-3 membrane binding and oligomerization encode negative feedback on PAR-3 dissociation. Second, membrane-bound PAR-3 promotes its own recruitment through a mechanism that requires the anterior polarity proteins CDC-42, PAR-6 and PKC-3. Using a kinetic model tightly constrained by our experimental measurements, we show that these two feedback loops are individually required and jointly sufficient to encode dynamically stable and locally inducible unipolar PAR-3 asymmetries in the absence of posterior inhibition. Given the central role of PAR-3, and the conservation of PAR-3 membrane-binding, oligomerization, and core interactions with PAR-6/aPKC, these results have widespread implications for PAR-mediated polarity in metazoa.

## Introduction

The PAR proteins are a highly conserved network of proteins that govern cell polarity in many different cells and tissues across the metazoa^1,2^. In all of these contexts, the PAR proteins become asymmetrically enriched in response to transient local cues. These asymmetries are reinforced through feedback loops in which PAR proteins locally promote or inhibit one another’s accumulation. Asymmetrically enriched PARs then act through different downstream targets to elaborate functionally polarized states. While the local polarizing cues vary widely across different contexts, the interactions among PAR proteins that govern the response to these cues is more highly conserved. Thus, a central challenge is to understand how systems of feedback among the PARs encode their ability to form and stabilize spatial patterns of enrichment that define functional polarity.

The one-cell *C. elegans* embryo (i.e. the zygote) has been a powerful model system for uncovering the core molecular circuitry that governs PAR polarity. During polarization of the zygote, two sets of PAR proteins become asymmetrically enriched in complementary anterior and posterior membrane domains. The anterior aPARs include the oligomeric scaffold PAR-3, the adaptor PAR-6, the kinase PKC-3, and the small GTPase CDC-42. The posterior pPARs include the kinase PAR-1, a *C. elegans*-specific protein called PAR-2, and a putative CDC-42 GAP called CHIN-1.

Polarization proceeds through two distinct phases, called establishment and maintenance^3^, which correspond respectively to mitotic interphase and mitosis. Before polarization, aPARs are uniformly enriched at the cell surface, while pPARs are largely cytoplasmic. During polarity establishment, a local sperm-derived cue triggers the redistribution of aPARs and pPARs into complementary domains. The sperm cue is closely associated with a centrosomal microtubule organizing center called the sperm MTOC that forms near the site of sperm entry^4,5^. The sperm MTOC acts through the aurora kinase AIR-1^6–9^ to trigger actomyosin-based cortical flows that transport aPARs towards the anterior pole^10,11^. In addition, astral microtubules associated with the sperm MTOC promote the local accumulation of pPARs PAR-1 and PAR-2 on the posterior membrane^12,13^.

**Figure 1.**
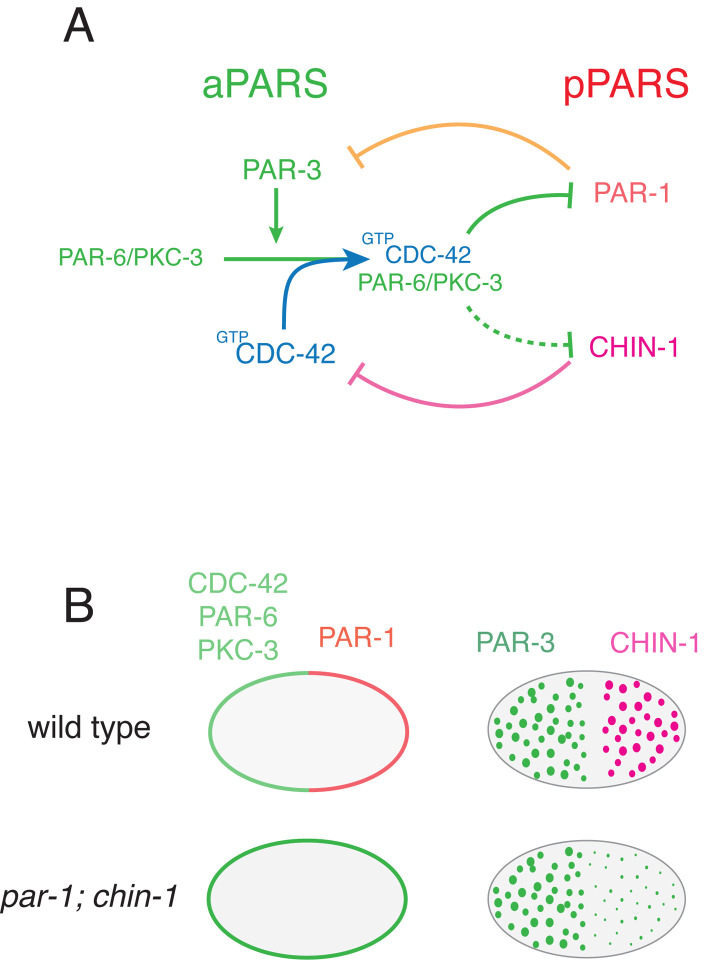
PAR polarity in the *C. elegans* embryo. **(A)** Schematic illustrating key interactions that maintain PAR asymmetries in the *C. elegans* zygote. On the left, PAR-3 promotes entry of the PAR-6/PKC-3 dimer into a complex with active CDC-42. Within this complex, PKC-3 inhibits cortical accumulation of posterior PARs. PAR-1 inhibits cortical accumulation of PAR-3, while CHIN-1 inhibits CDC-42 activity. **(B)** In embryos lacking posterior inhibitors PAR-1 and CHIN-1, CDC-42, PAR-6 and PKC-3 are uniformly distributed at the membrane, and all posterior PARs are absent or uniformly cytoplasmic. Nevertheless, PAR-3 oligomers remain asymmetrically distributed.

During mitosis, the centrosomes (thus the polarity cue) migrate to the cell center. However, PAR asymmetries are maintained through mitosis despite continuous diffusion and exchange of PAR proteins between the cytoplasm and the cell membrane^14,15^. The prevailing model is that PAR asymmetries are maintained by a network of mutually antagonistic interactions in which aPARs and pPARs act locally to inhibit one another’s accumulation (reviewed in Lang and Munro^2^, Figure 1A). Both PAR-3 and active CDC-42 are required for local recruitment of PAR-6/PKC-3 into an active complex with CDC-42^16,17^. Active PKC-3 inhibits local accumulation of pPARs, including PAR-1 and CHIN-1^12,17–22^. PAR-1 inhibits local accumulation of PAR-3^12,23^, while CHIN-1 inhibits local activation of CDC-42^17,22,24^. Because both PAR-3 and CDC-42 are required for local recruitment of PAR-6/PKC-3 during mitosis, either PAR-1 or CHIN-1 is sufficient to prevent posterior recruitment of PAR-6/PKC-3.

Strikingly, in zygotes that lack both PAR-1 and CHIN-1, all other pPARS are restricted to the cytoplasm, the aPARs CDC-42, PAR-6 and PKC-3 are uniformly enriched, but PAR-3 asymmetries still form and persist through mitosis^17^ (Figure 1B). PAR-3 is the keystone member of the PAR network without which all other PAR asymmetries are lost. Segregation of PAR-3 is a first step in polarization in many different contexts^12,25–28^. Thus, additional mechanisms must exist to maintain monopolar PAR-3 asymmetries in the absence of mutual antagonism with pPARs and these mechanisms must also contribute to shaping PAR asymmetries in normal embryos. Importantly, monopolar PAR-3 asymmetries have also been observed under wild-type conditions in other contexts, including in neuroblast stem cells^29–31^ and in mammalian hippocampal neurons^32^ and *Drosophila* male germline cells^33^. However, their molecular bases remain unknown.

A key feature of PAR-3 is its ability to form oligomers at the cell membrane. PAR-3 self-associates through a conserved N-terminal PB1-like domain, called the CR1 domain^34^. The purified CR1 domain assembles head to tail into helical filaments in vitro^35,36^. While PAR-3 contains multiple domains that can support direct binding to plasma membranes^37,38^, deletion or mutation of the CR1 domain prevents strong accumulation of PAR-3 in *C. elegans*^37^ and other systems^34,35,38^, and induces defects in embryonic polarity^16,37,39^. Oligomerization of PAR-3 plays an essential role in polarity establishment by coupling PAR-3 to the actomyosin cortex, facilitating segregation of PAR-3 and other aPARs by anterior-directed cortical actomyosin flows^16,26,39–41^. But it remains unknown whether oligomerization of PAR-3 plays additional roles in polarity maintenance, and in particular, whether and how oligomerization of PAR-3 might contribute to stabilizing PAR-3 asymmetries in the absence of mutual antagonism among the PARs.

Here, we combine single molecule imaging and particle tracking analysis with genetic manipulations and modeling to reveal a mechanism for self-stabilizing PAR-3 asymmetries in the *C. elegans* zygote. We show that PAR-3 asymmetries are maintained despite the continuous rapid exchange of subunits with PAR-3 oligomers. We find that two key factors maintain PAR-3 asymmetries in the absence of posterior inhibition: First, size-dependent membrane binding avidity of PAR-3 oligomers confers negative dependence of PAR-3 dissociation rate on local PAR-3 density – a form of positive feedback. Second, enhanced recruitment of PAR-3 to membranes where PAR-3 is already enriched, which requires the aPARs CDC-42, PAR-6 and PKC-3, provides a second form of positive feedback. Mathematical models, tightly constrained by our experimental measurements, show that the combination of negative feedback on dissociation and positive feedback on recruitment ensure the coexistence of two dynamically stable states: an unpolarized state, characterized by uniformly high levels of PAR-3, and a polarized state, characterized by asymmetric enrichment of PAR-3. The model predicts that transient local depletion of PAR-3, as occurs in response to the sperm cue during polarity establishment, can induce a transition between unpolarized and polarized states. Thus, self-stabilizing PAR-3 asymmetries encode a lasting memory of PAR distributions induced by a transient polarizing cue. Together, these results reveal a novel mechanism for stabilizing PAR-3 asymmetries in the absence of mutual antagonism, which depends on core dynamics of PAR-3 oligomer assembly and interactions with its conserved partners CDC-42, PAR-6 and PKC-3. We propose that similar mechanisms may underlie the stabilization of unipolar PAR asymmetries in other contexts.

## Results

### PAR-3 asymmetries approach a dynamic steady state during maintenance phase.

**Figure 2.**
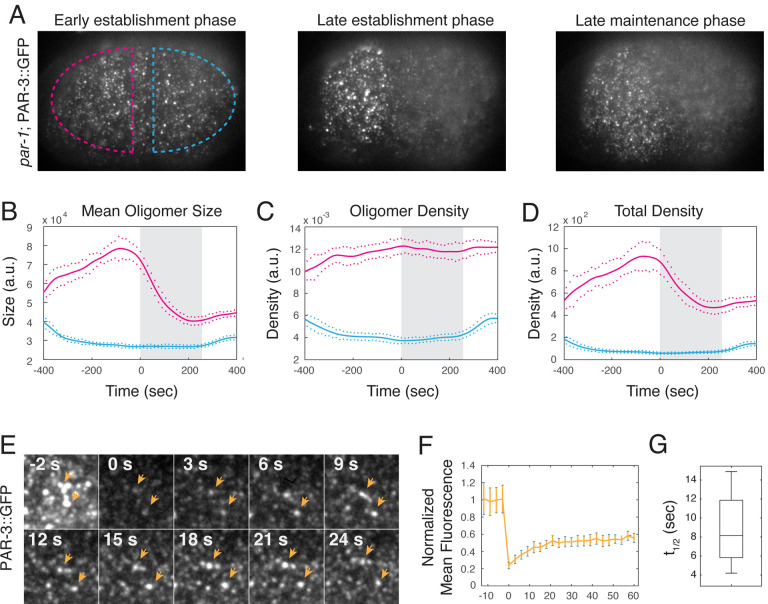
PAR-3 asymmetries approach a steady state during mitosis despite rapid continuous turnover. **(A)** Individual near-TIRF images from a time-lapse sequence showing PAR-3 oligomer intensities and distributions at the cell surface in homozygous *par-1* mutant embryos at early establishment, late establishment and late maintenance phase. **(B-D)** Results of particle detection analysis showing **(B)** mean oligomer size, **(C)** oligomer density, and **(D)** total PAR-3 fluorescence density over time on the anterior (magenta) and posterior (blue) membrane in homozygous *par-1* mutant embryos. Data are from n = 12 embryos aligned with respect to the onset of maintenance phase. The grey shaded area indicates maintenance phase. Solid lines indicate the mean and dots indicate S.E.M. at each time point. **(E)** Representative micrographs showing the time course of recovery of PAR-3::GFP in the anterior domain after photobleaching the entire field of view in an embryo in early maintenance phase. Timestamps indicate seconds after photobleaching. Orange arrowheads indicate individual PAR-3 oligomers that persist during recovery. **(F)** Plots of average fluorescence density vs time before and after photobleaching, normalized to prebleach levels and corrected for the overall decrease in fluorescence observed during maintenance phase (see Methods). Error bars indicate SEM (n = 4 embryos). **(G)** Box plot showing time to half maximal recovery (t_1/2_) after photobleaching.

As a first step to identifying mechanisms that maintain PAR-3 asymmetries in the absence of posterior inhibition by PAR-1, we used near-TIRF microscopy and single particle analysis to quantify dynamic changes in PAR-3 oligomer size and density during polarization in homozygous *par-1* mutant embryos (Figure 2A–D, Movie S1, see Methods). Consistent with previous reports, during polarity establishment, mean oligomer size, oligomer density, and overall density of PAR-3 increased steadily on the anterior cortex and decreased on the posterior cortex as cortical flows transport PAR-3 towards the anterior pole^39,41^. At the onset of maintenance phase, mean oligomer size and overall density of PAR-3 began to decrease, while oligomer density remained roughly constant. The rate of decrease was initially fast, but slowed progressively during maintenance phase, such that oligomer size and overall density approached plateaus at ~50% of their maximum values in late establishment phase. We observed similar PAR-3 oligomer dynamics in control (*par-1* heterozygote) embryos and throughout the cortex in embryos depleted of SPD-5 by RNAi, which lack a functional sperm cue and fail to establish polarity (Figure S1, Movie S1).

The slow approach to plateau levels of PAR-3 density and mean oligomer size could reflect either (a) the gradual disassembly of otherwise stable PAR-3 oligomers, or (b) the approach to a steady state governed by a dynamic balance of more rapid membrane-binding and oligomer-assembly kinetics. Consistent with the latter possibility, after photobleaching the entire cortex, total fluorescence in detected PAR-3 oligomers recovered to ~ 60% of the pre-bleach value with a mean half-time of 8.52 +\− 4.3 seconds (Figure 2F; n = 4 embryos; Movie S2). Direct inspection of the recovery time series suggests that individual PAR-3 oligomers recover fluorescence on similarly fast timescales (orange arrowheads in Fig 2E). These data suggest that PAR-3 asymmetries approach a steady state that is maintained in the face of more rapid exchange of cytoplasmic PAR-3 with the membrane and with membrane-bound oligomers.

### Intrinsic kinetics of membrane binding and oligomerization produce an approximately exponential distribution of PAR-3 oligomer sizes.

**Figure 3.**
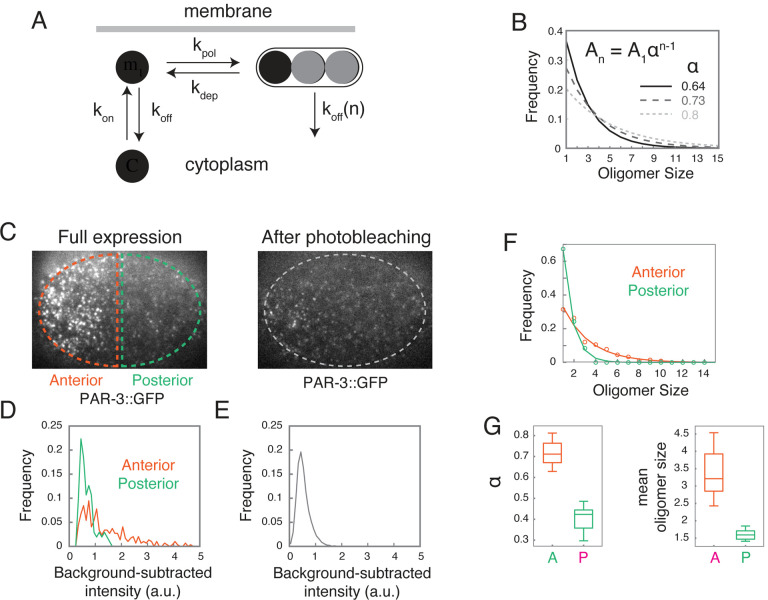
PAR-3 oligomer size distributions reflect kinetics of membrane binding and polymerization. **(A)** A simple kinetic model for PAR-3 oligomerization dynamics. Monomers bind reversibly to the membrane with rate constants *k*_*on*_ and *k*_*off*_. Membrane-bound monomers bind oligomers with rate constants *k*_*pol*_ and *k*_*dep*_ and oligomers dissociate from the membrane with size-dependent rate constant *k*_*off*_(*n*) where *n* is the number of subunits in the oligomer. We neglect direct binding of cytoplasmic monomers to membrane bound oligomers and membrane binding of oligomers with size n > 1 (see text for justification). **(B)** Predicted steady state distribution of oligomer sizes *A*_*n*_ = *A*_1_*α*^*n*–1^ for different values of oligomerization strength *α* (see text for details) **(C)** Micrographs showing distributions of PAR-3 before (left panel) and after 30 seconds of continuous laser exposure at full power (right panel) in late maintenance phase *par-1* mutant embryos. Dashed orange and green outline the regions in which anterior and posterior measurements were made. **(D)** Distributions of anterior (orange) and posterior (green) speckle intensities measured in a representative embryo. **(E)** Distributions of speckle intensities after photobleaching (gray) in the same embryo. **(F)** Inferred distributions of PAR-3 oligomer sizes on the anterior and posterior cortex of a representative embryo, based on calibrating raw intensities to the estimated intensity distribution of single molecules. Dashed lines indicate data fit to the equation *A*_*n*_ = *A*_1_*α*^*n*–1^. **(G)** Estimates of oligomerization strength *α* (left) and mean oligomer size s (right) on the anterior and posterior of n = 8 individual embryos in late maintenance phase.

To probe the mechanisms underlying this dynamic steady state, we used single molecule imaging and particle tracking analysis to characterize the kinetics of PAR-3 membrane binding and oligomerization in relation to slowly changing distributions of PAR-3 oligomers. As a framework for this analysis, we considered a simple kinetic model (Figure 3A, Modeling Supplement) in which: (a) cytoplasmic PAR-3 monomers bind reversibly to the plasma membrane, (b) membrane-bound PAR-3 monomers form small oligomers, and (c) oligomers dissociate from the membrane with size-dependent kinetics – i.e. with an off rate that decreases with oligomer size. For reasons justified later (see Modeling Supplement and below), we assume that direct binding of cytoplasmic monomers to PAR-3 oligomers, and binding of cytoplasmic PAR-3 oligomers to the membrane can both be neglected.

**Figure 4.**
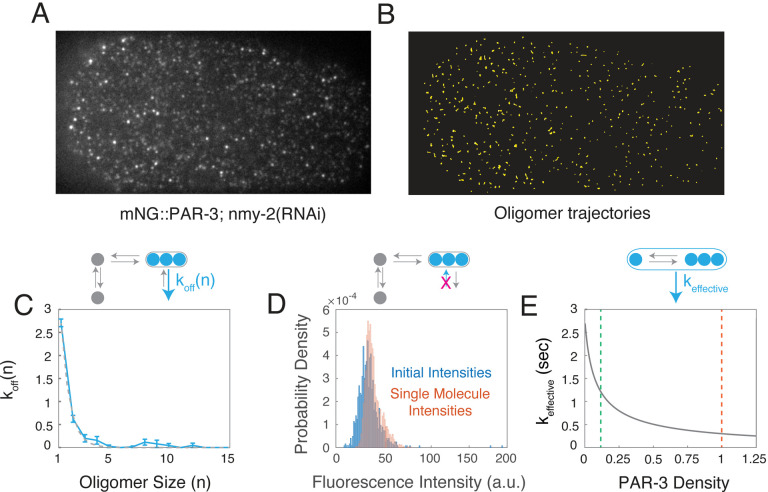
Size-dependent dissociation of PAR-3 oligomers induces negative feedback on average rates of PAR-3 dissociation. **(A)** Representative micrograph of a maintenance phase embryo expressing endogenously-tagged mNeonGreen::PAR-3 and depleted of myosin II heavy chain NMY-2 by RNAi. **(B)** Individual trajectories (yellow traces) for all oligomers detected in the first movie frame. **(C)** Estimated rate constants for dissociation rates of PAR-3 oligomers from the membrane as a function of inferred number of subunits n. Error bars represent the SEM. Data were compiled from n = 3 individual embryos. **(D)** Overlay of the distributions of background-subtracted intensities of (blue) newly detected PAR-3::GFP speckles from the data in (A) and (peach) single molecule speckles after photobleaching in the same cells. **(E)** Predicted dependence of the effective rate constant for PAR-3 dissociation $k_{\text{effective}}=\frac{\sum_{n=1}^{\infty }\;k_{off}(n)n\alpha^{n-1}}{\sum_{n=1}^{\infty }\;n\alpha^{n-1}}$ on the total density of membrane-bound PAR-3, normalized to anterior density at steady state (see Methods for details).

We focused initially on measuring the intrinsic kinetics of PAR-3 membrane binding and oligomerization to confirm the model’s core assumptions, constrain kinetic parameters, and compare key model predictions to experimental observations. If oligomers dissociate much more slowly than monomers, as we confirm below (see Figure 4C), then the steady state distribution of oligomer sizes should be approximately exponential, given by *A*_*n*_~ *A*_1_*α*^*n*–1^, where *A*_*n*_ is the density of oligomers with n subunits, $\alpha =\frac{k_{pol}}{k_{\text{dep}}}A_{1}$ measures oligomerization strength, and the mean oligomer size is $s=\frac{1}{1-\alpha }$. Moreover, both oligomerization strength and mean oligomer size should increase with density of membrane-bound PAR-3 (see Modeling Supplement for details).

To test these predictions, we measured the distributions of PAR-3 oligomer sizes on anterior and posterior membranes in *par-1* mutant embryos expressing endogenously-tagged PAR-3::GFP at quasi-steady state during late maintenance phase. We used the intensities of single PAR-3::GFP molecules, measured in the same embryos after photobleaching, as an internal size standard (Figure 3C–E, see Methods). Consistent with prediction, both the anterior and posterior distributions of oligomer sizes were well-fit by single-exponential functions (Figure 3F). The estimated oligomerization strengths (*α*_*A*_ = 0.730 ± 0.052, *α*_*P*_ = 0.424 ± 0.054) and mean oligomer sizes (*s*_*A*_= 3.48 ± 0.65, *s*_*p*_ = 1.65 ± 0.16) were higher on the anterior cortex where total PAR-3 density is higher (Figure 3G). Moreover, given the measured A:P ratio of PAR-3 densities, the ratio of oligomerization strengths (or mean oligomer sizes) agreed closely with model predictions. We also confirmed this relationship through a systematic variation of PAR-3 densities (see Figure 7D and text below). Thus, the distributions of PAR-3 oligomer sizes are consistent with a quasi-steady state governed by reversible membrane binding and polymerization.

### Density-dependent oligomer size and size-dependent oligomer dissociation confers positive feedback on PAR-3 accumulation.

If mean oligomer size increases with PAR-3 density and oligomer dissociation rate decreases with size, then our model predicts that the effective dissociation rate constant for PAR-3 oligomers, defined as:

$$k_{\text{effective}\;}=\frac{\sum_{n=1}^{\infty }\;\;k_{\text{off}}(n)n\alpha^{n-1}}{\sum_{n=1}^{\infty }\;\;n\alpha^{n-1}}$$

should decrease with increasing oligomer size. This represents a form of positive feedback of PAR-3 on its own accumulation, whose strength depends on self-affinity of PAR-3 $\left(\frac{k_{\text{pol}}}{k_{\text{dep}}}\right)$ and the size-dependence of oligomer dissociation *k*_*off*_(*n*). To measure the size-dependent rate of oligomer dissociation *k*_*off*_(*n*) during maintenance phase, we used streaming acquisition and particle tracking analysis of PAR-3 endogenously tagged with mNeonGreen (mNG::PAR-3), using the mean intensities of single molecules to calibrate estimates of oligomer size, as described above (see Methods). To improve the accuracy of particle tracking, we performed these experiments in embryos depleted of the myosin II heavy chain NMY-2 by RNAi, which increases the distance between particles by preventing the segregation of PAR-3 to the anterior pole during polarity establishment and eliminates the movements of particles driven by local actomyosin contractility (Figure 4A,B; Movie S3) To avoid the confounding effects of photobleaching, we used the intensities of oligomers in the first frame of the imaging sequence to estimate oligomer size n, and the fraction of oligomers in each size class with bound lifetimes > 1 sec to estimate *k*_*off*_(*n*) (see Methods). Our measurements suggest that *k*_*off*_(*n*) decreases sharply with oligomer size, becoming negligible for oligomer sizes > 3 subunits (Figure 4C). Using the same data to compare the intensities of PAR-3 oligomers arriving at the membrane with single molecule intensities, we also confirmed that PAR-3 recruitment is dominated by monomer binding (Figure 4D).

To quantify the strength of positive feedback due to size-dependent oligomer dissociation, we constrained the model using our estimates of both density-dependent oligomerization strengths (Figure 3G) and size-dependent dissociation *k*_*off*_(*n*) (Figure 4C), and then computed the dependence of the effective dissociation rate constant *k*_*effective*_ on PAR-3 density at steady state (Figure 4E). Given the measured A:P ratio of PAR-3 densities, the model predicts a 3.8-fold A:P ratio in *k*_*effective*_. Thus, positive feedback on PAR-3 accumulation, due to size-dependent oligomer dissociation kinetics, accounts for roughly half of the 8-fold asymmetry in PAR-3 observed in late maintenance phase.

### Rapid membrane binding and slower oligomerization underlies the maintenance of PAR-3 asymmetries.

To further constrain our model’s predictions, we used single molecule approaches to measure the kinetics of membrane unbinding and depolymerization. Based on the above results (Figure 2E,F; Figure 3G; Figure 4C), and previous work^17,26,39^, we expect that most anterior PAR-3 molecules are associated with stably-bound slow-moving PAR-3 oligomers, and therefore their disappearance should reflect the slow kinetics of depolymerization. On the other hand, we expect that most posterior PAR-3 molecules are freely diffusing monomers, whose disappearance reflects the faster kinetics of membrane unbinding.

**Figure 5.**
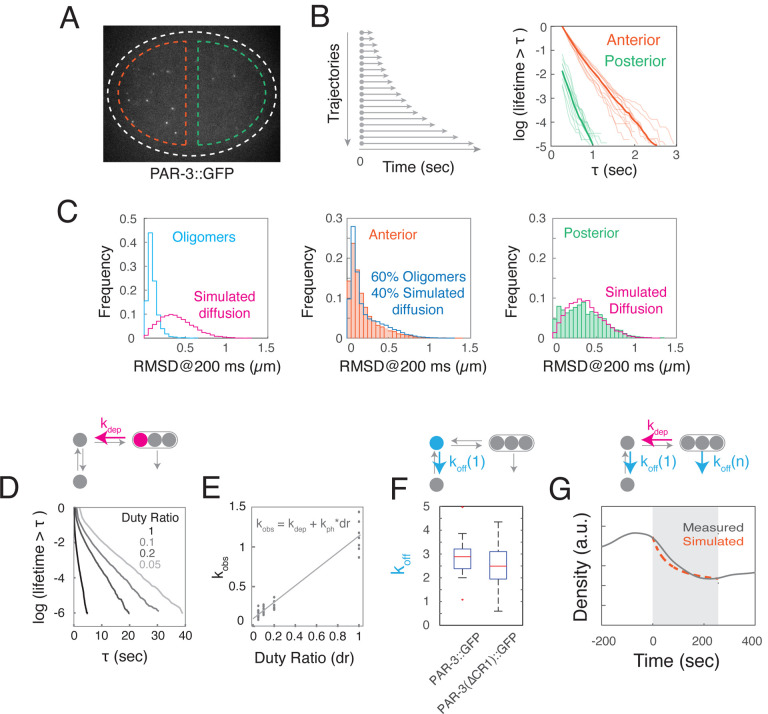
Membrane unbinding and depolymerization set the timescale for slow approach to steady state distributions of PAR-3 during maintenance phase **(A)** Representative image showing single molecules of PAR-3::GFP imaged at high laser power and short exposure times to ensure accurate tracking of fast diffusing molecules. **(B)** (left) Schematic illustrating the scheme for constructing release curves. (right) Release curves measured for single molecules of PAR-3::GFP on the anterior and posterior cortex of wild type embryos under continuous illumination. Thin traces show release curves for individual embryos (n = 8); Thick traces show data summed for all embryos. Data were normalized by total numbers of anterior tracks. **(C)** Distributions of root mean square displacements after 200 msec (RMSD_200_) for anterior (orange) and posterior (green) molecules pooled from 8 embryos. Plots on the right show reference RMSD@200 distributions for (cyan) tracked PAR-3 oligomers (n = 7 embryos) and (magenta) simulated Brownian diffusion with diffusivity D = 0.1 μm^2^/sec. In middle panel, the blue curve shows a weighted (60/40) sum of RMSD@200 distributions for oligomers and for simulated diffusion. **(D)** Release curves for PAR-3::GFP molecules imaged with fixed laser power and exposure times but different duty ratios. Each curve represents the sum of data from multiple embryos: (Duty ratio = 1, n = 7; Duty ratio = 0.2; n = 7, Duty ratio = 0.1; n = 12; Duty ratio =, n = 13). **(E)** Disappearance rate constants (*k_obs_*) measured for different duty ratios by fitting the linear portions of the release curves (see Methods for details). The dashed line represents a linear least-squares fit of the data to the equation *k*_*obs*_ = *k*_*dep*_ + k_*pb*_ * *dr*. **(F)** Estimates of *k*_*off*_ from streaming data for full length PAR-3::GFP (n = 9 embryos) and oligomerization defective PAR-3(ΔCR1)::GFP (n = 11 embryos). **(G)** Comparison of measured (blue curve) approach to steady state and the simulated approach (dashed red curve) given measured values for *k*_*off*_(*n*) and *k*_*dep*_.

To confirm these expectations, we imaged single molecules of endogenously-tagged PAR-3::GFP using high laser power and streaming acquisition to ensure accurate particle tracking (Figure 5A;Movie S4; see Methods). We quantified mobilities by measuring the distribution of root mean square displacements after 200 msec (RMSD@200; Figure 5B), and disappearance rates by constructing release curves plotting the numbers of molecules with lifetimes > t as a function of t (Figure 5C).

Consistent with our expectations, we found that posterior PAR-3 molecules have RMSD@200 distributions that are indistinguishable from those produced by simulated Brownian diffusion at ~0.1 μm^2^/sec (Figure 5B right), while anterior PAR-3 molecules have RMSD_200 distributions that are well-fit by the superposition of RMSD@200 distributions measured for PAR-3 oligomers and for Brownian diffusion (Figure 5B left). Similarly, comparison of release curves revealed that anterior molecules disappear more slowly than posterior molecules, confirming that membrane unbinding is much faster than depolymerization, although photobleaching obscures the magnitude of this difference (Figure 5C). Therefore, we used the release kinetics of anterior PAR-3 molecules to estimate the rate constant for depolymerization (*k*_*dep*_), and release kinetics of posterior PAR-3 molecules to estimate the rate constant for monomer unbinding (*k*_*off*_).

To measure *k*_*dep*_, we used RNAi against GFP to reduce expression of transgenic PAR-3::GFP to single molecule levels. We held the unit exposure constant (fixed laser power and 50 msec exposure time), varied the duty ratio of exposure (*dr*), and fit the resulting release curves with single exponentials (Figure 5E) to estimate a rate constant for disappearance *k*_*diss*_, which reflects both depolymerization (*k*_*dep*_) and photobleaching (*k*_*pb*_). Plotting *k*_*diss*_ vs *dr*, and fitting the data to *k*_*diss*_ = *k*_*dep*_ + *k*_*pb*_ * *dr* (Figure 5E), we obtained estimates of the rate constants for photobleaching (*k*_*pb*_ = 1.04 +/− 0.04/s) and depolymerization (*k*_*dep*= = 0.08 +/−0.02/) (see Methods), which agrees well with the halftime for recovery of fluorescence after photobleaching (Figure 2G).

To measure the monomer unbinding rate *k*_*off*_, we fit release curves for single molecule data collected in streaming mode to estimate rate constants for anterior and posterior disappearance $k_{\text{diss}}^{\text{ant}}$ and $k_{\text{diss}}^{\text{post}}$ (Figure 5C, orange and green curves). We used $k_{\text{diss}}^{\text{ant}}$ and our measurement of *k*_*dep*_ above to estimate $k_{pb}=k_{\text{diss}}^{\text{ant}}-k_{\text{dep}}$. Then, we used $k_{\text{diss}}^{\text{post}}$ and k_pb_ to measure the monomer unbinding rate $k_{\text{off}}=k_{\text{diss}}^{\text{post}}-k_{\text{pb}}=2.58+/-0.3/\text{sec}$ (Figure 5F). Importantly, this estimate agrees well with the value measured above (k_off_(1) = 2.7/sec; Figure 4C). We measured a similar value for k_off_ using a transgenically-expressed version of PAR-3 that lacks the N terminal CR1 oligomerization domain (PAR-3(ΔCR1)::GFP; Figure 5F, Figure S2C–D, Movie S4).

These measurements show that depolymerization is ~ 30-fold slower than membrane unbinding of PAR-3 monomers, but fast enough to explain the continuous exchange of fluorescent PAR-3 within oligomers during maintenance phase. Because the flux of monomers into and out of oligomers must balance at steady state, these measurements sharply constrain the possible contribution of direct binding of cytoplasmic PAR-3 monomers to membrane-bound oligomers. The maximum fraction of monomer recruitment due to direct binding is ~17%, although the actual value is likely to be significantly lower (see Modeling Supplement), justifying our choice to neglect direct binding in our simple model.

Finally, we used our measurements of mean oligomer size, *k*_*off*_(*n*), and *k*_*dep*_ to predict the overall kinetics of approach to steady state, assuming that binding kinetics are constant throughout maintenance phase. We observed good agreement between simulated and measured dynamics (Figure 5G), confirming that the intrinsic kinetics of PAR-3 membrane binding and oligomerization set the timescale of approach to steady state during maintenance.

### Asymmetric monomer recruitment contributes to stabilizing PAR-3 asymmetries during maintenance phase.

We showed above that positive feedback due to size-dependent oligomer dissociation accounts for only half of the ~8-fold asymmetry in PAR-3 density observed at steady state in *par-1* mutant embryos. Moreover, theoretical analysis shows that, regardless of strength, this form of feedback is insufficient to stabilize an asymmetric distribution of membrane-bound oligomers (see Lang and Munro, 2022^42^).Thus we wondered whether positive feedback on PAR-3 recruitment could account for the missing ~2-fold AP difference in PAR-3 densities, and if so, whether the combined effects of both forms of feedback would be sufficient to ensure the dynamic stability of PAR-3 asymmetries in the absence of posterior inhibition by PAR-1.

**Figure 6.**
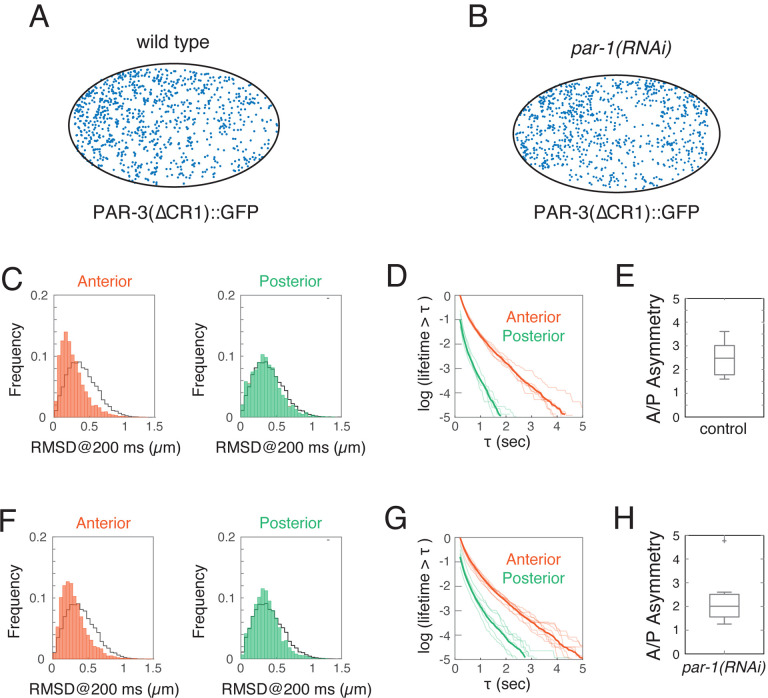
Asymmetric recruitment of PAR-3 monomers underlies maintenance of PAR-3 asymmetries. **(A,B)** Distribution of single molecule appearance events for oligomerization-defective PAR-3(ΔCR1)::GFP in representative **(A)** control and **(B)** *par-1(RNAi)* embryos, detected by single molecule imaging. Each dot represents a single binding event. **(C-E)** RMSD@200 distributions **(C)**, release curves **(D)**, and estimates of recruitment asymmetry **(E)** measured from pooled single molecule trajectories for n = 6 control embryos. **(F-H)** RMSD@200 distributions **(F)**, release curves **(G)**, and estimates of recruitment asymmetry **(H)** measured from pooled single molecule trajectories for n = 7 *par-1(RNAi)* embryos.

To test these possibilities, we first used fast single molecule imaging to measure rates of single molecule recruitment on anterior and posterior membranes during maintenance phase in wild-type and *par-1(RNAi)* embryos (Figure 6A,B; Movie S5). To avoid the confounding effects of direct binding of cytoplasmic PAR-3 to membrane-bound oligomers, we performed these measurements on a variant of PAR-3 (PAR-3(ΔCR1)::GFP lacking the conserved N-terminal CR1 domain that mediates homo-oligomerization^34,35,37^. In these experiments, single molecule speckles that appear *de novo* on the membrane are rapidly photobleached, allowing us to detect and count many appearance events per unit area and time. To avoid counting cytoplasmic molecules that diffuse into the focal plane, we focused on molecules with tracked lifetimes >= 200 msec, for which distributions of RMSD@200ms matched those expected for membrane-bound PAR-3 molecules undergoing Brownian diffusion (Figure 6C,D; green histograms). Extrapolating from release curves for these molecules (Figure 6E,G; see Methods), we estimated the total number of membrane binding events per unit area and time for anterior and posterior molecules. We found that during maintenance, single PAR-3(ΔCR1)::GFP molecules bound the anterior membrane 2.5 times more frequently than the posterior membranes in wild-type embryos and 2.0 times more frequently in *par-1(RNAi)* embryos (Figure 6F,H). Thus PAR-3 monomers bind membranes at higher rates where PAR-3 is already enriched, consistent with positive feedback on PAR-3 recruitment. Together, the A:P ratios in effective dissociation rate (3.8) and recruitment rate (~2) are sufficient to account quantitatively for the total PAR-3 asymmetry observed at late maintenance phase in *par-1* mutant embryos.

**Figure 7.**
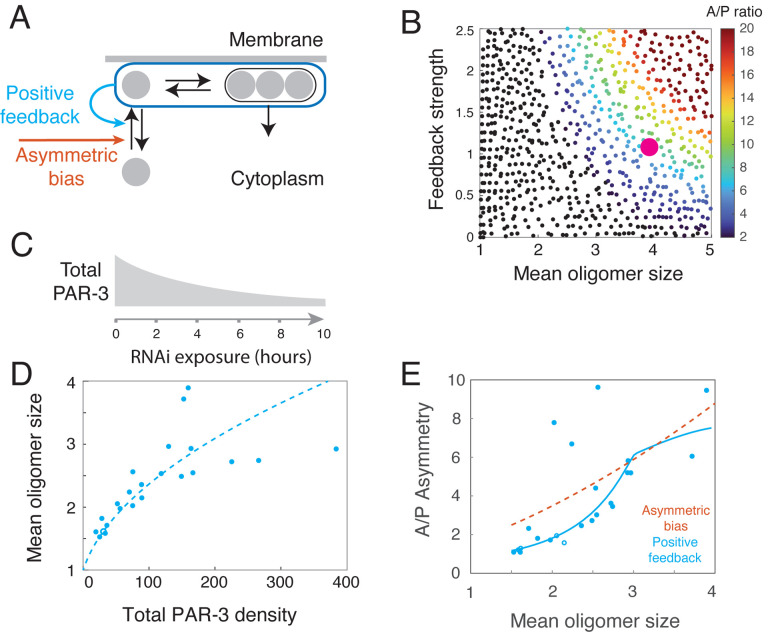
Positive feedback on PAR-3 monomer recruitment and size-dependent dissociation of oligomers, are both necessary and sufficient to stabilize PAR-3 asymmetries. **(A)** Modified versions of the reaction diagram in Figure 3A, showing two alternative assumptions for the mechanism that underlies asymmetric recruitment: (blue arrow): Membrane-bound PAR-3 feeds back to promote recruitment of PAR-3 monomers to the membrane. (orange arrow): A fixed asymmetric input, whose strength is independent of PAR-3 densities, biases recruitment to the anterior cortex. **(B)** Phase plane derived for the version of the model with positive feedback on membrane recruitment, showing how the ability to polarize depends on mean oligomer size and feedback strength (see text for details). Experimental measurements of mean oligomer size and feedback strength in the *C. elegans* zygote place PAR-3 in the region of phase space where polarization is possible (large magenta dot). **(C)** Schematic illustrating the PAR-3 depletion experiment. Total pool of PAR-3 can be tuned by varying feeding time on RNAi plates. **(D)** Scatter plot showing the relationship between mean oligomer size and total membrane-bound PAR-3 measured across increasing levels of PAR-3 RNAi depletion. Solid curve shows a fit of the data to model prediction. **(E)** Scatter plot showing the relationship between mean oligomer size and PAR-3 asymmetry during late maintenance phase. Filled dots represent embryos where polarity was established by late interphase and unfilled dots represent embryos where polarity was not established. The magenta curve represents the predicted relationship for the model in which asymmetric recruitment is the result of a cryptic bias and the cyan line represents the prediction of a model where it is the result of saturating positive feedback (see text for details).

We then asked: If the observed membrane binding asymmetry reflects positive feedback on PAR-3 recruitment, is this feedback sufficiently strong to ensure dynamically stable PAR-3 asymmetries in the absence of posterior inhibition? We added a simple form of feedback to the model (cyan arrow in Figure 7A), in which the rate constant for monomer recruitment is given by:

$$k_{\text{on}}=\left(k_{\text{basal}}+k_{f}f(A)\right)$$

where *k*_*basal*_ is the rate constant for basal recruitment, *f*(*A*) defines the form of positive feedback, and *k*_*f*_ tunes its strength. Because recruitment asymmetries of PAR-3 are similar in early and late maintenance phase, despite a nearly two-fold change in the anterior PAR-3 density, we assumed a simple form of saturating feedback governed by:

$$f(A)=\text{min}\left(A,A^{\left(\text{Sat}\;\right)}\right)$$

where *A*^(*Sat*)^ is the density of PAR-3 above which feedback saturates. To facilitate direct comparisons with experimental measurements, we defined a feedback strength as the ratio of the recruitment rate at the spatially uniform steady state to the basal recruitment rate:

$$S_{\text{feedback}}=\frac{k_{f}A^{\left(\text{Sat}\right)}}{k_{\text{basal}}}$$

With these assumptions, the model predicts one of two dynamical behaviors: either (a) no stably polarized state exists, (i.e. the spatially uniform steady state is always stable) or (b) spatially uniform and asymmetric stable states coexist, and a transient local depletion of PAR-3 can induce a transition from the spatially uniform to the polarized state, as observed during normal polarity establishment. We refer to this latter case as inducible polarity. As shown in the phase diagram in Figure 7B, the predicted dynamics depend on just two quantities: the mean oligomer size *s*, which measures the strength of positive feedback due to size-dependent oligomer dissociation and *S*_*feedback*_, the strength of positive feedback on monomer recruitment defined above. The model predicts that both forms of feedback are required for stable inducible polarity and identifies the combinations of feedback that would be sufficient to produce the stable PAR-3 asymmetry observed at steady state during maintenance phase.

To locate the maintenance phase embryo in this phase diagram, we parameterized the model by first assuming a reasonable target for *A*_*h*_, the anterior steady state density of PAR-3 as a function of the total available density. We then chose a value *A*^(Sat)^ < *A*_*h*_ for the saturation level, in accordance with our observation that feedback is saturated during maintenance phase. To fix the polymerization kinetics, we used our empirically measured values for *k*_*dep*_ and $k_{\text{off}}^{\left(n\right)}$, then varied *k*_pol_ so that polymerization kinetics agreed with our experimental data. This left the two binding parameters *k*_basal_ and *k*_*f*_, which we set to the unique values that reproduced the target steady state density *A*_*h*_ and A/P asymmetry in total PAR-3 density. The large magenta dot locates the model-predicted behavior within the phase diagram in Figure 7B for the inferred parameter values. This choice of parameters yielded a recruitment asymmetry within the range measured experimentally (Fig. 6H), providing evidence for the accuracy of our model. Importantly, the predicted value of feedback strength $\frac{k_{f}A^{\left(\text{Sat}\right)}}{k_{\text{basal}}}$ was relatively insensitive to the exact choice of *A*^(*Sat*)^ and *A*_*h*_. This analysis reveals that for tightly constrained model parameters, the combined strength of feedback due to size-dependent oligomer dissociation and feedback on monomer recruitment is sufficient to endow the zygote with the ability to form dynamically stable PAR-3 asymmetries in response to a transient local input (pink dot in Figure 7B).

The asymmetric membrane binding of PAR-3(ΔCR1)::GFP is consistent with positive feedback of PAR-3 on its own recruitment (cyan arrow in Figure 7A). However, it could also reflect an asymmetric bias on monomer binding that does not depend on local densities of PAR-3 (orange arrow in Figure 7A). To distinguish these two scenarios, we used simulations to predict how the PAR-3 asymmetry should vary with total PAR-3 levels (and thus mean oligomer size), given either a fixed asymmetric bias or positive feedback on recruitment. We then tested these predictions, by varying PAR-3 levels in living embryos (Figure 7C–E).

We found that a key difference between the two scenarios lies in the PAR-3 asymmetries predicted for low levels of total PAR-3 (compare cyan and orange curves in Figure 7E). For a fixed two-fold A:P bias in monomer binding, the predicted PAR-3 asymmetries persist as the total PAR-3 approaches 0, approaching a fixed value > 1.7 (orange curve in Figure 7E), which is determined by the combined effects of asymmetric recruitment and the negative dependence of effective dissociation rate on density of membrane-bound PAR-3. In contrast, for positive feedback on membrane recruitment whose strength yields a two-fold difference in recruitment rates at steady state, our simulations predict that PAR-3 asymmetries disappear entirely below a threshold level of PAR-3 (cyan curve in Figure 7E).

To distinguish these possibilities, we used RNAi against PAR-3 to systematically vary the total abundance of PAR-3 in the system. We used a strain expressing endogenously tagged PAR-3::GFP, allowing us to measure the total density of membrane associated PAR-3, the mean oligomer size, and the asymmetry in PAR-3 in late maintenance phase for each embryo (see Methods for details). We focused our analysis on embryos that established PAR-3 and we verified that loss of asymmetry during maintenance was not caused by redistribution of PAR-3 by cortical flow (Figure S3). We confirmed the predicted dependence of mean oligomer size on total density of membrane-bound PAR-3 (Figure 7D). More importantly, plotting the observed PAR-3 asymmetry against mean oligomer size shows that the response to progressive depletion of PAR-3 strongly resembles, both qualitatively and quantitatively, the response predicted for the positive feedback scenario (Figure 7E). We conclude that asymmetric recruitment of PAR-3 to the cell membrane results from positive feedback, and that this positive feedback on recruitment, combined with positive feedback due to size-dependent dissociation of PAR-3 oligomers, is sufficient to support stable PAR-3 asymmetries during maintenance in embryos lacking posterior inhibition by PAR-1.

### PAR-6/PKC-3 are required for positive feedback on PAR-3 recruitment and stable PAR-3 asymmetries.

Because the asymmetric recruitment of PAR-3 does not require an intact CR1 domain, positive feedback on recruitment must involve the interaction of PAR-3 with additional factors. The other anterior polarity proteins PAR-6, PKC-3 and CDC-42 are an attractive set of candidates. PAR-6 and PKC-3 form an obligate heterodimer that can bind directly to either PAR-3 or CDC-42^43–50^. The PAR-6/PKC-3 heterodimer is thought to cycle between PAR-3-bound and CDC-42-bound states during polarization^16,39,51,52^. Although the bulk of PAR-6/PKC-3 is thought to be complexed with CDC-42 during maintenance phase, its local recruitment to the membrane requires both PAR-3 and CDC-42^17,51^, suggesting that PAR-6/PKC-3 interacts at least transiently with PAR-3. Therefore, we hypothesized that PAR-6/PKC-3, and perhaps CDC-42, might act as intermediaries to promote positive feedback on PAR-3 recruitment.

**Figure 8.**
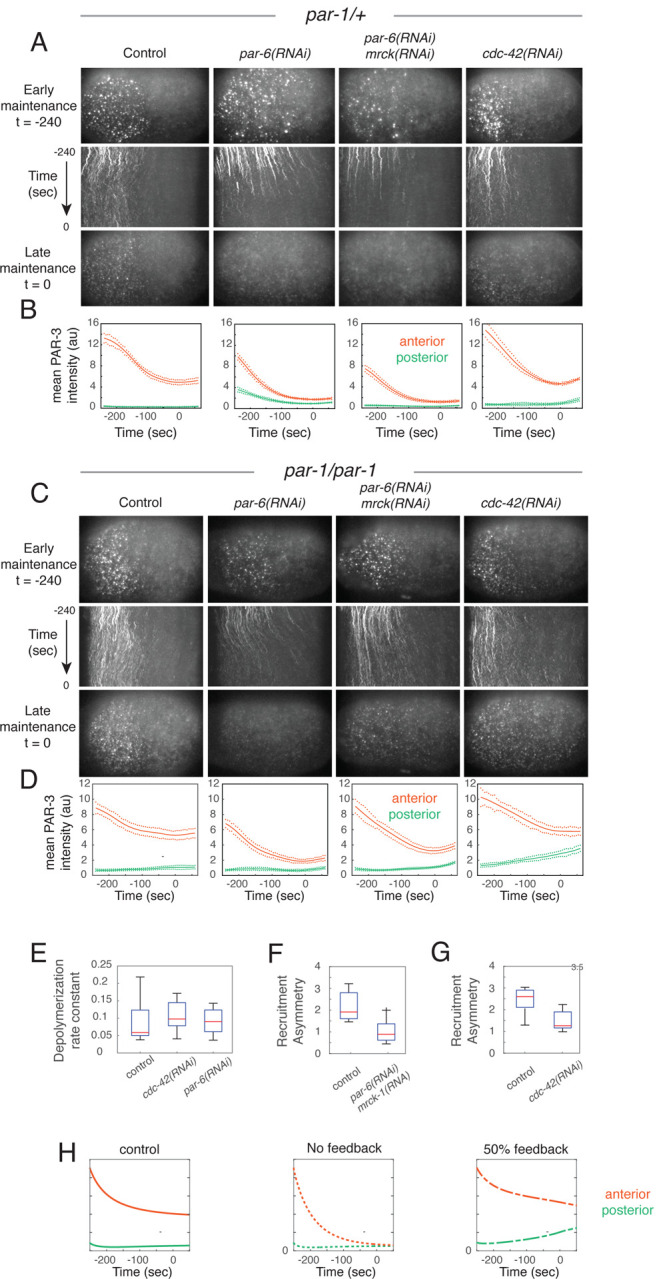
CDC-42 and PAR-6/PKC-3 reinforce PAR-3 asymmetries during maintenance phase by promoting asymmetric recruitment of PAR-3 **(A)** Representative micrographs showing PAR-3 oligomer sizes and distribution during maintenance phase in *par-1* heterozygote (*par-1/+*) embryos for the indicated RNAi depletions. Top and bottom micrographs show the distribution of PAR-3 oligomers in early (−240 sec) and late (0 sec) maintenance phase. Time is measured relative to the point where anterior density of PAR-3 reached a minimum in late maintenance phase (see Methods for details). Kymographs show how the distribution changes over the 240 seconds between these time points. Diagonal streaks in the kymographs indicate transport of PAR-3 oligomers by cortical flow. **(B)** Corresponding plots of total fluorescence density of PAR-3 within segmented speckles during maintenance phase. Bold lines indicate the mean and red dots indicate the standard error of the mean across individual embryos (control: n = 8, *cdc-42(RNAi)*: n = 7, *par-6(RNAi):* n = 6, *par-6(RNAi)*; *mrck-1(RNAi*): n = 8). **(C)** Representative micrographs showing PAR-3 oligomer sizes and distribution during maintenance phase in *par-1* homozygous (*par-1/par-1*) embryos for the indicated RNAi depletions. Data are presented as described in **(A)**. **(D)** Corresponding plots of total fluorescence density of PAR-3 within segmented speckles during maintenance phase. Bold lines indicate the mean and red dots indicate the standard error of the mean across individual embryos (control: n = 10, *cdc-42(RNAi)*: n = 6, *par-6(RNAi):* n = 9, *par-6(RNAi)*; *mrck-1(RNAi*): n = 14). **(E)** Estimates of depolymerization rates (*k*_*dep*_) in wild type (n = 8), *cdc-42(RNAi)* (n = 12) and *par-6(RNAi)* (n = 14) embryos expressing transgenic PAR-3::GFP. **(F)** Boxplots showing A:P ratio of rates (#/unit area) for binding of oligomerization-defective PAR-3 molecules to the cell membrane in control (n = 6) and *par-6(RNAi)*; *mrck-1(RNAi*) (n = 6) embryos in early maintenance phase. **(G)** Boxplots showing A:P ratio of rates (#/unit area) for binding of oligomerization-defective PAR-3 molecules to the cell membrane in control (n = 6) and *cdc-42(RNAi)* (n = 6) embryos in late maintenance phase. **(H)**. Simulations showing predicted changes in anterior and posterior PAR-3 densities during maintenance phase starting from the initial distributions observed at the onset of maintenance phase. Left panel: Control conditions; Middle panel: Feedback strength set to zero to mimic *par-6(RNAi)*; *mrck-1(RNAi*); Right panel: basal recruitment rate and feedback strength adjusted to mimic *cdc-42(RNAi)*.

To evaluate this possibility, we examined the effects of depleting PAR-6 on PAR-3 asymmetries during polarization in *par-1* mutant and heterozygous control embryos. Consistent with previous reports^11,16,53^, PAR-3 oligomers accumulate on the membrane during polarity establishment phase in control (*par-1/+*) embryos depleted of PAR-6, although cortical flows and anterior enrichment of PAR-3 were significantly reduced (Figure 8A; *par-6(RNAi)*, Movie S6). However, these asymmetries were completely lost during maintenance phase. This loss of asymmetry was accompanied by both a rapid decrease in PAR-3 density and oligomer size on the anterior cortex, and by the rapid transport of PAR-3 oligomers towards the posterior pole by aberrant posterior directed cortical flows^17^ (Figure 8A, Movie S6). However, co-depleting PAR-6 and MRCK-1 (which is required for cortical contractility and flow during mitosis^17,22^) produced a similarly rapid decrease in anterior oligomer size and complete loss of PAR-3 asymmetry during maintenance phase (Figure 8A,B; *par-6(RNAi);mrck-1(RNAi)*, Movie S6).

The decrease in PAR-3 density and oligomer sizes produced by depleting PAR-6/PKC-3 in *par-1* heterozygotes could be due to increased inhibition by PAR-1^12^, which accumulates uniformly in embryos lacking PAR-6/PKC-3^54^. However, depleting PAR-6 in *par-1* homozygous mutant embryos produced similarly rapid and complete loss of PAR-3 oligomers during maintenance phase (Figure 8C,D; *par-6(RNAi)*, Movie S7). We also observed rapid decrease in PAR-3 density, oligomer size and overall PAR-3 asymmetry in *par-1* homozygotes co-depleted of PAR-6 and MRCK-1 relative to untreated *par-1* homozygotes, although the loss was incomplete at late maintenance phase (Figure 8C,D; *par-6(RNAi);mrck-1(RNAi*, Movie S7)). Altogether these data show that PAR-6/PKC-3 play a central role in maintaining PAR-3 asymmetries during maintenance phase independent of posterior inhibition by PAR-1 and cortical flow.

The changes in PAR-3 density and oligomer size induced by co-depleting PAR-6 and MRCK-1 could be caused by increased depolymerization or monomer dissociation, decreased recruitment rates, or both. Using single molecule imaging at low duty ratios as above (Figure 5E), we found there was no significant difference in depolymerization rates (*k*_*dep*_) during early maintenance phase in PAR-6-depleted embryos compared to untreated controls (Figure 8E; Figure S4A). Similarly, comparing release curves for PAR-3(ΔCR1)::GFP suggests that there is no significant difference in the dissociation rates of monomeric PAR-3 in control vs *par-6(RNAi); mrck-1(RNAi)* embryos (Figure S4B,C). In contrast, the asymmetric recruitment of PAR-3(ΔCR1)::GFP during early maintenance phase was abolished in embryos co-depleted of PAR-6 and MRCK-1 (Figure 8F), even though endogenous PAR-3 is highly enriched on the anterior cortex during early maintenance phase in these embryos (Figure 8A,B). Thus PAR-6/PKC-3 stabilize PAR-3 asymmetries during maintenance phase by promoting asymmetric recruitment of PAR-3.

Finally, we used the empirically constrained model to assess whether the loss of PAR-3 recruitment asymmetry produced by co-depleting PAR-6 and MRCK is sufficient to explain the dynamic loss of PAR-3 asymmetries observed during maintenance phase in these embryos. We set *k*_*f*_ = 0 to mimic complete loss of asymmetric binding, and simulated PAR-3 dynamics from the initial distribution measured at late establishment phase in *par-1* homozygote embryos co-depleted of PAR-6 and MRCK (Figure 8C,D; *par-6(RNAi);mrck-1(RNAi)*). As expected from the phase diagram on Figure 7B, the model predicts the complete loss of asymmetry, but the time course is too slow to reach completion during maintenance phase, consistent with the incomplete loss of asymmetry observed at late maintenance phase in these embryos (Figure 8H). We conclude that PAR-6/PKC-3 act as a key intermediary to support positive feedback of PAR-3 on its own recruitment and dynamically stable PAR-3 asymmetries.

### CDC-42 promotes dynamically stable PAR-3 asymmetries by tuning basal rates of PAR-3 recruitment

We also assessed the consequences of depleting CDC-42 in *par-1* mutant and heterozygous control embryos. In *par-1* heterozygotes depleted of CDC-42, PAR-3::GFP asymmetries formed during polarity establishment phase and relaxed toward lower levels during maintenance phase, as observed in controls (Figure 8C; control, *cdc-42(RNAi)*, Movie S6), although overall density and mean oligomer size were slightly higher than in controls (Figure 8D; compare control and *cdc-42(RNAi)*). There was no significant difference in the depolymerization of full length PAR-3::GFP or dissociation of monomeric PAR-3(ΔCR1)::GFP relative to controls (Figure 8E, Figure S4D,E).

In contrast, asymmetric recruitment of PAR-3(ΔCR1)::GFP was reduced, but not abolished in embryos depleted of CDC-42 (Figure 8F), even though endogenous PAR-3 remains asymmetrically enriched during maintenance phase in these embryos, compared to controls (Figure 8A,B). In *par-1* homozygotes depleted of CDC-42, PAR-3:GFP became enriched during polarity establishment phase to slightly higher levels than observed in untreated homozygous controls, but these asymmetries were almost completely lost during polarity maintenance phase (Figure 8C,D; *cdc-42(RNAi)*, Movie S7). Strikingly, while loss of PAR-3 asymmetry in *par-6(RNAi);mrck-1(RNAi)* embryos is associated with a decrease in anterior PAR-3 density and oligomer size, the loss of asymmetry in *cdc-42(RNAi)* embryos was associated with an increase in posterior PAR-3 density and oligomer size (Figure 8D; *cdc-42(RNAi)*, Movie S7).

The observation that posterior densities and oligomer sizes increase, while anterior densities and oligomer sizes remain the same in embryos depleted of CDC-42 suggest that the reduced asymmetry is primarily due to an increase in the basal recruitment rate *k*_*basal*_, which would cause a decrease in the overall feedback strength measured by $S_{\text{feedback}}=\frac{k_{f}A^{\left(\text{Sat}\right)}}{k_{\text{basal}}}$. To test this possibility, we modified our model by reducing *k*_*basal*_ to 50% of the control value while holding *k*_*f*_ constant to approximate the recruitment asymmetry measured in CDC-42-depleted control embryos. Then we simulated PAR-3 dynamics from the initial distribution measured at late establishment phase in *par-1* homozygote embryos depleted of CDC-42 (Figure 8C,D; *cdc-42(RNAi)*). The simulations reproduced the qualitative signature and time course of PAR-3 asymmetry loss observed in CDC-42-depleted embryos in which anterior PAR-3 levels fall toward and posterior levels rise toward an intermediate spatially uniform level (Figure 8H). Again, the loss of asymmetry was incomplete at late maintenance phase, as observed experimentally (Figure 8H).

In summary, these data show that both PAR-6/PKC-3 and CDC-42 are required for dynamically stable PAR-3 asymmetries in the absence of posterior inhibition by PAR-1, but the data suggest that PAR-6/PKC-3 and CDC-42 act in different ways: PAR-6/PKC-3 is required for positive feedback on PAR-3 recruitment, while CDC-42 reduces basal rates of recruitment, which increases the overall strength of positive feedback.

## Discussion

### Two feedback loops encode robust self-stabilizing unipolar PAR-3 asymmetries

Studies of PAR-mediated polarity have focused on how mutual antagonism between two sets of PAR proteins drive their stable enrichment in complementary cortical domains^12,17,23,55–58^. However, the ability of PARs to form and stabilize *unipolar* asymmetries has remained poorly understood^29–33^. Here we have combined single molecule analysis with mathematical modeling and experimental perturbations to identify two positive feedback loops that synergistically ensure robust dynamically stable unipolar PAR-3 asymmetries in *C. elegans* zygotes. First, the intrinsic dynamics of PAR-3 membrane binding, oligomerization, and size-dependent oligomer dissociation encode negative feedback on PAR-3 dissociation. Second, PAR-3 acts indirectly to promote its own recruitment through a mechanism that depends on its conserved binding partners PAR-6 and PKC-3. We have shown that these two modes of positive feedback are individually necessary and jointly sufficient to account, quantitatively, for the locally inducible and dynamically stable PAR-3 asymmetries observed in zygotes lacking posterior inhibition by PAR-1. Thus, a tight correspondence between model-predicted and observed dynamics, integrating multiple independent measurements of single molecule kinetics, oligomer size distributions and the time evolution of cell-scale asymmetries, reveals a robust, internally self-consistent and quantitively accurate picture of how self-stabilizing unipolar PAR-3 asymmetries emerge from molecular scale kinetics.

### Intrinsic dynamics of membrane binding and oligomerization encode nonlinear positive feedback on PAR-3 accumulation

Self-oligomerization is a conserved feature of PAR-3^34^ Recent studies in *C. elegans* zygotes have emphasized how this property can contribute to the advective transport of PAR-3 (and its binding partners PAR-6/PKC-3) during symmetry-breaking, through increased physical coupling to the cortical actomyosin cytoskeleton and through increased residence time via size-dependent binding avidity^17,26,39–41^. Here we have shown that the intrinsic dynamics of membrane binding and oligomerization also confer non-linear positive feedback on PAR-3 accumulation. Combining single molecule analysis and kinetic modeling, we find that three processes account quantitatively for the distribution of PAR-3 oligomers, and the time scale of approach to steady state during maintenance phase: fast monomer binding (*k*_*off*_ = ~3 *sec*), slower oligomerization (*k*_*dep*_= ~ 0.1 *sec*), and even slower size-dependent dissociation of PAR-3 oligomers. Our model predicts, and our experiments confirm, a positive dependence of mean oligomer size on density, and we have measured a sharp decrease in oligomer dissociation rate with size. Together these make the average rate constant for PAR-3 dissociation a sharply non-linear function of PAR-3 density. For measured kinetic parameters, we find that this negative feedback on PAR-3 dissociation contributes a nearly 4-fold A:P difference in the flux of PAR-3 molecules off the membrane, accounting for half the total asymmetry observed in *par-1* mutant embryos lacking posterior inhibition of PAR-3.

Importantly, non-linear feedback on dissociation is a generic consequence of reversible membrane binding and oligomerization. In this simple generic form, it does not consume energy, and therefore it cannot alone sustain dynamically stable asymmetries. However, it can sharply reduce the amount of additional energy-consuming feedback needed for stable asymmetries. Because weak membrane binding and self-oligomerization are widespread and easily evolved properties of proteins, we expect that many types of cells use this form of feedback to promote symmetry-breaking and stable polarity. Indeed, our results add to a growing body of work emphasizing the importance of clustering/oligomerization of peripheral membrane binding proteins to establishment and maintenance of cell surface asymmetries in polarized cells^17,59–61^.

### PAR-6/PKC-3 mediate positive feedback on PAR-3 recruitment

Our observations suggest that the additional feedback required for dynamically stable PAR-3 asymmetries come from positive feedback on PAR-3 monomer recruitment. First, monomeric PAR-3(ΔCR1)::GFP binds at > 2-fold higher rates to anterior membranes where PAR-3 itself is enriched. In principle this binding asymmetry could reflect either a fixed PAR-3-independent bias, or positive feedback on monomer recruitment. However, comparing model variants tightly constrained by our experimental measurements can cleanly distinguish these possibilities. A model based on fixed bias cannot explain the observed dependence of PAR-3 asymmetry on total PAR-3 density, while a model based on simple feedback can and does for experimentally measured parameters.

Our data suggest that the other aPARs play central roles in mediating (PAR-6/PKC-3) or tuning (CDC-42) positive feedback on PAR-3 recruitment. Co-depleting PAR-6 and MRCK-1 (to prevent aberrant cortical flows) in *par-1* mutants leads to rapid loss of PAR-3 asymmetry during maintenance phase, from an initially polarized state. This loss is not due to changes in depolymerization or monomer dissociation rates; instead, co-depleting PAR-6 and MRCK-1 completely blocks the asymmetric recruitment of PAR-3(ΔCR1)::GFP, even though endogenous full-length PAR-3 remains highly asymmetric in these embryos. Thus PAR-6/PKC-3 are required for positive feedback on PAR-3 monomer recruitment and dynamically stable unipolar PAR-3 asymmetries in the absence of posterior inhibition by PAR-1. Depleting CDC-42 in *par-1* mutant embryos also leads to loss of PAR-3 asymmetries, but through an increase in basal rates of PAR-3 monomer recruitment and aberrant posterior accumulation of PAR-3. In both cases, changing a single quantity – the strength of positive feedback on recruitment – allows the model predicts correctly the mode and time course of asymmetry loss observed in experiments.

The mechanism(s) by which PAR-6/PKC-3 and CDC-42 mediate positive feedback on PAR-3 recruitment remain unclear, but previous studies and our data constrain the class of possible mechanisms. PAR-3 asymmetries persist during maintenance phase in *par-1;chin-1* double mutant embryos in which CDC-42 activity, PAR-6 and PKC-3 are uniformly enriched^17^. Thus, positive feedback does not require the asymmetric enrichment of PAR-6/PKC-3 or active CDC-42. Instead, these data favor a mechanism in which a transient interaction of PAR-6/PKC-3 with PAR-3 oligomers licenses their ability to recruit additional PAR-3 monomers from the cytoplasm. A similar mechanism was previously proposed for positive feedback on GTPase activation by Anillin^62^.

PAR-6/PKC-3 heterodimers can form distinct ternary complexes with PAR-3 and active CDC-42^16,51,52^. PAR-3 and CDC-42 compete for binding to PAR-6/PKC-3 in vitro^63^; PAR-6/PKC-3 dimers are thought to cycle between PAR-3 and CDC-42-bound states in vivo^16^, and binding to both PAR-3 and CDC-42 is required for localization of PAR-6/PKC-3 during maintenance phase in C. elegans zygotes^17,51^. Thus, one possibility is that PAR-6/PKC-3 heterodimers act as a shuttle – cycling between PAR-3 and CDC-42 bound states; In particular, CDC-42 may bind and displace PAR-6/PKC-3 when it is bound to PAR-3 and cytoplasmic PAR-3 monomers may bind and displace PAR-6/PKC-3 when it is bound to CDC-42. For this to work, an enzymatic step would be required to toggle the relative affinities of PAR-6/PKC-3 for CDC-42 and PAR-3, but since both PAR-3 and CDC-42 are both direct phosphorylation substrates of PKC-3^64,65^, there are multiple ways in which this mechanism could work. One caveat is that this scenario does not explain why depleting CDC-42 leads to increased basal rates of PAR-3(ΔCR1)::GFP binding.

An alternative scenario is that PAR-6/PKC-3 dimers bound to PAR-3 could recruit a second PAR-3 molecule. Strong binding of PAR-6/PKC-3 to PAR-3 in vitro, and membrane recruitment of PAR-6/PKC-3 *in vivo* requires direct binding between a PDZ-binding motif (PBM) on PKC-3 and the PDZ2 domain of PAR-3^50^. However, a PAR-6/PKC-3 heterodimer can also bind PAR-3 via PAR-6^48^, or through an interaction between the kinase domain of PKC-3 and PAR-3^50,66^. Thus, in principle, PAR-6/PKC-3 heterodimers complexed with PAR-3 at the membrane could recruit additional molecules of cytoplasmic PAR-3. For this to constitute positive feedback, an energy consuming step (e.g phosphorylation of PAR-3 by PKC-3) would be required. Moreover, the fast mobilities of newly-bound PAR-3(ΔCR1)::GFP molecules imply that their asymmetric recruitment does not require direct stable binding to existing PAR-3 oligomers. Thus, a bridging mode of recruitment would have to be very transient, delivering cytoplasmic monomers into a freely diffusing membrane-bound state, or it would have to preference the use of membrane-bound PAR-3 monomers over oligomers. Distinguishing these and other possible mechanisms for PAR-6/PKC-3 mediated feedback on PAR-3 monomer recruitment will be an important goal for future work.

PAR-3 is the keystone member of the PAR polarity network, which is required for all other PAR asymmetries. In the *C. elegan*s zygote, PAR-3 is the only member that remains asymmetric when all other known members of the PAR network are absent or symmetrically distributed^17^. In the *C. elegans* zygote, and other cells, the spatial distribution of PAR-3 determines where PAR-6/PKC-3 load onto the membrane and into a complex with active CDC-42, to form a gradient of PKC-3 activity that defines anterior (or apical) identity and opposes posterior (or basolateral) identity^16,17,67^. Thus, the spatial distribution of PAR-3 dictates polarity, even in cells where mutual inhibition plays an important role

Our analysis of a model tightly constrained by empirical measurements reveals that the combination of negative feedback on PAR-3 dissociation and positive feedback on PAR-3 recruitment encodes the co-existence of two dynamically stable states: a spatially uniform state with high PAR-3 levels as observed during maintenance phase in unpolarized zygotes lacking a functional sperm cue; and a polarized state with high anterior and low posterior levels of PAR-3, matching those observed during maintenance phase in polarized zygotes. Importantly, the model predicts that local depletion of PAR-3, mimicking the effects of cortical flow or local PAR-1 accumulation during zygotic symmetry breaking^11,12^, can induce an irreversible transition from the spatially uniform to the stably polarized state. Thus, the intrinsic kinetics of PAR-3 membrane-binding and oligomerization, plus weak feedback on PAR-3 recruitment mediated by PAR-6/PKC-3, endow *C. elegans* zygotes with a robust symmetry-breaking response to the sperm cue, independent of mutual antagonism. Because these core features of PAR-3 and its interactions with PAR-6/PKC-3 are conserved across the metazoa, it seems likely that they may contribute to stabilizing unipolar asymmetries observed in other contexts, for example in neuroblasts^27,31^, or meiotic oocytes^68^, and that they may play key roles in controlling PAR asymmetries, even in cells like the *C. elegans* zygote that also rely on mutual inhibition.

## STAR Methods

### C. elegans culture and RNAi

We cultured *C. elegans* strains under standard conditions^69^ on 60mm petri plates containing *E. coli* bacteria (OP50) raised on normal growth medium (NGM). Table 1 provides a list of strains used in this study. Unless otherwise specified, strains were provided by the Caenorhabditis Genetics Center, which is funded by the National Center for Research Resources.

We performed RNAi experiments using the feeding method^70^. We obtained bacteria targeting *par-1*, *par-3*, *par-6*, *cdc-42*, *mrck-1*, *spd-5*, and *nmy-2* from the Kamath RNAi library^71^, and bacteria targeting GFP from Jeremy Nance. Briefly, we grew bacteria to log phase in LB with 50μg/ml ampicillin, seeded ~300μl of culture onto NGM plates supplemented with 50μg/ml ampicillin and 1mM IPTG, incubated plates at room temperature for two days, and then stored them at 4°C for up to one week before use. We transferred L4 larvae or young adults (for *par-3(RNAi*)) to feeding plates and then cultured them at 25°C before imaging as follows: 0 – 8 hours at 25°C for *par-3(RNAi*) and 24 – 48 hours at room temperature (21–23°C) for all others, adjusting exposure time to achieve the desired phenotype. We verified strong depletions of SPD-5 by observing complete failure of polarity establishment, strong depletions of PAR-6 and CDC-42 by observing symmetric first and second cleavages, and strong depletions of NMY-2 and MRCK-1 by observing absence of cortical flows during polarity establishment and/or maintenance phases.

### Live imaging

For single molecule imaging experiments, we mounted embryos in egg salts containing ~21 *μm* diameter polystyrene beads. For all other experiments, we mounted embryos in eggs salts on agarose pads.

For data reported in Figures 3, 4, 5D–F, 6, 7, S2, S4, and S5, we used an Olympus IX50 inverted microscope equipped with an Olympus OMAC two-color TIRF illumination system, a CRISP autofocus module (Applied Scientific Instrumentation), and a 1.49 NA oil immersion TIRF objective. Laser illumination at 488 nm from a 50-mW solid-state Sapphire laser (Coherent) was delivered by fiber optics to the TIRF illuminator. We magnified images by 1.6x and collected them on an Andor iXon3 897 EMCCD camera, yielding a pixel size of 100 nm. Image acquisition was controlled by Andor IQ software. For the data reported in Figures 2, 5A–C&G, 8, S1 and S3, we used a Nikon Ti2-E inverted microscope equipped with a LunF XL laser combiner housing solid state 488nm, 561nm and 640nm lasers and feeding three separate TIRF illumination arms, a Ti2 N-ND-P Perfect Focus unit, and a CFI APO 100X 1.49NA TIRF lens. We magnified images by 1.5X and collected them on an Andor IXON Life 897 EM-CCD camera to achieve a pixel size of ~106nm. Image acquisition was controlled by Nikon Elements software.

For all experiments, we used near-TIRF illumination. We set the laser illumination angle to standard values that we chose empirically to approximately maximize signal-to-noise ratio while maintaining approximately even illumination across the field of view. All embryos were oriented with their AP axis perpendicular to the axis of TIRF illumination to avoid any confounding effects of illumination gradients in comparisons of anterior vs posterior PAR-3 dynamics. Further details of the imaging conditions used for specific quantitative analyses appear below.

### Image analysis

We performed particle detection and tracking as previously described^15,17^. Briefly, we used a slightly modified Matlab implementation (http://people.umass.edu/kilfoil/ downloads.html) of methods introduced by Crocker and Grier^72^ to detect diffraction-limited features of interest as local intensity peaks in bandpass filtered images and then to determine their centroids and background-subtracted integrated intensities. We use μTrack software (http://github.com/DanuserLab/u-track) developed by Jaqaman and colleagues^73^ to track features over time. We integrated particle detection and tracking and all subsequent analyses using custom MATLAB scripts that are available upon request.

### Timelapse analysis of PAR-3 oligomer dynamics

We measured PAR-3 oligomer dynamics in a *par-1* mutant strain expressing a C-terminal fusion of GFP to PAR-3 from the endogenous locus (hereon *par-1*; PAR-3::GFP), using *par-1/+* heterozygotes as controls. We collected images using near-TIRF illumination in timelapse mode using 10% laser power and 50msec exposure times at 3 sec intervals to minimize photobleaching. For each embryo and each timepoint, we identified PAR-3 clusters within hand-drawn anterior and posterior regions of interest (ROIs). For this and all subsequent measurements of feature intensities, we measured background-subtracted intensities by measuring integrated intensity within a circular ROI with radius of 3 pixels (300 nm) around the feature’s centroid position and using the mean intensity within an annulus of width 2 pixels surrounding the central ROI to estimate local background. We then determined the minimal polygon containing all detected features and used its area to compute the densities of features or total PAR-3 fluorescence. For *spd-5(RNAi)* embryos, we used a single ROI for all measurements. For individual embryos, we plotted the smoothed mean intensities vs time to determine the timepoint in late establishment phase when mean intensities began to fall sharply, which coincided roughly with the onset of pseudocleavage (PC) furrow relaxation. Then we used this timepoint to align data across multiple embryos. We then computed means and standard errors across embryos from un-normalized data.

### Measurements of PAR-3 oligomer size distribution

We measured the distribution of PAR-3 oligomer sizes during late maintenance phase in homozygous *par-1*; PAR-3::GFP mutant embryos. We first identified embryos in establishment phase before pseudocleavage. We used 30% laser power and 50 msec exposures at 1 sec intervals to focus on the cortex while minimizing photobleaching. Following pseudocleavage relaxation, we waited for 4 minutes and then acquired images in streaming mode for 50 seconds using 100% laser power and a 50 msec exposure time. For each embryo, we used hand-drawn ROIs to separate anterior and posterior features. we pooled all features detected in the first three frames and used these to estimate the distribution of PAR-3::GFP oligomer intensities. During the last 20 seconds of continuous exposure to 100% laser power, the distribution of background-subtracted feature intensities approximated a Gaussian distribution, as described previously^15^. We took the mean of a gaussian fit to this distribution as our estimate of single molecule intensities.

We used three different approaches to estimate the distribution of PAR-3 oligomer sizes. In the first approach, based on Bayesian inference, we initially assumed each feature had an equal prior probability of containing between 1 and 20 subunits. We then calculated the posterior probabilities that an observed feature contained n subunits (0 < n <=20) given the conditional probabilities of an n-mer producing the observed fluorescence intensity, assuming that means and variances for the distributions of n-mer intensities were equal to the mean and variance of the single molecule distribution multiplied by n. Then we used these posterior probabilities to update the prior probabilities, repeating until the prior and posterior probability distributions converged, yielding an estimate of the steady-state size distribution. In a second approach, based on maximum likelihood estimation, we assumed that the distribution of feature intensities equals a weighted sum of the distributions of n-mers of size 1 to 20, determining the distributions of nmer intensities as described above. We then used the lsqnonlin function in MATLAB to compute the most likely values for each of the weights. Finally, as a third approach, we binned feature by intensity, assigning all feature with intensities in the interval [(n – 0.5)*I_sm, (n+0.5)*I_sm)] to size bin n, where I_sm is the mean intensity of single GFP molecules. Because all three approaches produced very similar results, we used the third (simplest) approach to produce the size distributions shown in Figure 3.

### Measuring single molecule disappearance rates and mobilities

To measure single molecule mobilities and disappearance rates, as in Figure 5 A–C&G, and Figure S2, we imaged single PAR-3::GFP molecules in near-TIRF mode using streaming acquisition with 50 msec exposure times and 100% laser power. For some experiments, we used brief (< 5 sec) pre-exposure to 100% laser power in widefield mode to reduce the density of labeled molecules. During the first ~ 5 sec of imaging, the surface density of labeled molecules decreased rapidly to single molecule levels due to photobleaching and then decreased slowly over several minutes of observation. We collected single molecule data after the initial photobleaching interval and performed particle tracking analysis as described above. For each embryo, we used hand-drawn ROIs to separate anterior and posterior trajectories. To quantify single molecule mobilities, for all trajectories with lifetime >= 200 msec, we computed the root mean square displacements for a fixed interval (t = 200 msec). Then we plotted histograms for trajectories pooled over all embryos. To compute reference histograms for oligomers, we imaged endogenously-tagged PAR-3::GFP at the same frame rates, but with lower laser power (10%), performed tracking analysis and histogram construction as described above. To construct reference histograms for simulated Brownian diffusion, we simulated 10,000 particles undergoing Brownian diffusion with D = 0.1 μm^2^/sec, added gaussian noise with mean 0 and SD = 50 nm to approximate localization errors in our system^15^, then performed tracking analysis and histogram construction as described above.

To estimate rate constants for monomer dissociation, we first estimated rate constants for disappearance of single PAR-3::GFP molecules ($k_{\text{diss}}^{\text{ant}}$ and $k_{\text{diss}}^{\text{post}}$) as follows: For each embryo, we constructed release curves for anterior and posterior trajectories by plotting the number of trajectories with lifetime > t as a function of time. Plotted on a logarithmic scale, these release curves are approximately linear (i.e. they approximate exponential decay) for t > t_min_, where t_min_ is different for anterior and posterior release curves. We used non-linear least squares (with Matlab’s built-in ***fit*** function) to fit the release curves to $x(t)=x_{0}e^{-k_{\text{diss}}t}t>t_{min}$, using t_min_ = 200 msec for posterior release curves and t_min_ = 0.5 sec for anterior release curves.

To infer monomer dissociation rates k_off_ from $k_{\text{diss}}^{\text{ant}}$ and $k_{\text{diss}}^{\text{post}}$, we used $k_{\text{diss}}^{\text{ant}}$ and our measurement of *k*_*dep*_ (see below) to estimate $k_{pb}=k_{\text{diss}}^{\text{ant}}-k_{\text{dep}}$. Then, we used $k_{\text{diss}}^{\text{post}}$ and k_pb_ to measure the monomer unbinding rate $k_{\text{off}}=k_{\text{diss}}^{\text{post}}-k_{\text{pb}}$. To estimate dissociation rates for monomeric PAR-3 (PAR-3(CR1)::GFP, we imaged single molecules of PAR-3 (PAR-3(CR1)::GFP under the same conditions used for PAR-3::GFP, measured $k_{\text{diss}}^{\text{post}}$ as described above, and used the estimate of k_pb_ from PAR-3::GFP to infer $k_{\text{off}}=k_{\text{diss}}^{\text{post}}-k_{\text{p}}$.

### Measuring depolymerization rates

To measure depolymerization rates *k*_*dep*_, we used a transgenic strain expressing PAR-3::GFP. We used RNAi against GFP followed by photobleaching to reduce the density of transgenic PAR-3::GFP to single molecule levels as described previously^15^. We then imaged single PAR-3 molecules using 100 % laser power and 50 msec exposures, holding photobleaching rate *k*_*pb*_ (thus total photobleaching during each exposure) constant, while varying the duty ratio of exposure (dr). For each duty ratio, we performed single particle tracking and constructed release curves as described above. We fit the release curves to exponential functions of the form $x(t)=x_{0}e^{-k_{\text{obs}}t}$, using t_min_ = 1 sec to exclude the more rapid (2.7/sec) monomer dissociation. We then used MATLAB’s built-in ***fit*_**function to obtain a linear least squares fit to *k*_*obs*_ = *k*_*dep*_ + *dr* * *k*_*pb*_. We took the y-intercept and slope of the linear fits as our estimates of *k*_*dep*_ and *k*_*pb*_, respectively, and report the confidence intervals returned by the ***fit*** function.

To compare depolymerization rates in control, *par-6(RNAi);mrck-1(RNAi)* and *cdc-42(RNAi)* embryos, we imaged all embryos for 120 seconds during early maintenance phase with a 5% duty ratio, and a fixed unit exposure (50 msec at 100% laser power). We constructed release curves and measured *k*_*obs*_ for each embryo as described above using t_min_ = 2 sec. Then we used the of *k*_*pb*_ from above to estimate *k*_*dep*_ = *k*_*obs*_ − *dr* * *k*_*pd*_ for each embryo.

### Measuring oligomer size-dependent dissociation.

To measure oligomer size-dependent dissociation rates, we imaged embryos expressing NeonGreen::PAR-3 in streaming acquisition mode using 30% laser power and 50 N’L exposure times during maintenance phase of embryos depleted of NMY-2. We performed particle tracking on the first 20 frames of each image sequence to avoid confounding effects of photobleaching on oligomer size inference. We inferred the size of each tracked particle based on its initial intensity using the single molecule distribution as a standard, as described above. For each inferred oligomer size n, we computed the fraction of oligomers *F*_*n*_ that remained after 1 sec. Assuming a single-rate first-order dissociation reaction, we solved $F_{n}=e^{-k_{off}(n)}$ to estimate *k*_*off*_(*n*) = −ln (*F*_*n*_).

### Measuring asymmetric binding of monomeric PAR-3

To measure the relative binding rates of cytoplasmic PAR-3(ΔCR1)::GFP molecules to anterior and posterior membranes, we first photobleached PAR-3(ΔCR1)::GFP molecules by exposing embryos to 100% laser power in widefield illumination mode for 5–10 seconds. We then imaged single PAR-3::GFP molecules with near-TIRF illumination using streaming acquisition with 100% laser power and 50 msec exposure times. We performed single particle tracking as described above and used hand-drawn ROIs to select anterior and posterior trajectories. We constructed the release curves as described above for trajectories with lifetimes >= 200 msec to avoid counting spurious trajectories. To estimate the total number of trajectories with lengths < 200 msec that represent membrane binding events, we used the slope of the release curve at t = 200 msec to extrapolate from t = 200 to t = 0. Finally, we used the areas of ROIs to normalize estimates of total number and used these normalized quantities to determine the ratio of anterior to posterior binding rates for each embryo.

### PAR-3 depletion experiments

To obtain partial depletion of PAR-3, we placed young adult worms on RNAi feeding plates for 2–8 hours. To assess whether PAR-3 polarity was established prior to the onset of maintenance phase and whether posterior-directed cortical flows redistribute PAR-3 oligomers during maintenance phase, we imaged embryos in timelapse mode using 50 msec exposures and 30% laser power at 2.5–5 second intervals for 4 minutes following pseudocleavage relaxation. Immediately following this period, we imaged the same embryos in streaming mode with 100% laser power and 50 msec exposures for 50 seconds, and performed feature detection and oligomer size inference as described above. We determined the fluorescence density of segmented PAR-3 features in anterior and posterior ROIs and computed the anterior-posterior asymmetry of PAR-3 as the ratio of these values.

### FRAP experiments

We performed FRAP experiments using the following protocol: We collected 10 images with 50 msec exposures using near-TIRF illumination at 1 second intervals using 30% laser power. We then subjected embryos to constant near-TIRF illumination with 100% laser power for 1.5 seconds to photobleach molecules at the cell surface. We then tracked recovery in timelapse mode with 50 msec exposures at 3 second intervals using 30% laser power. Because PAR-3 levels decrease during early maintenance phase, we normalized measurements of fluorescence during recovery using unbleached control data collected over the same timeframe.

## Supplementary Material

Supplement 1 **Movie S1. Dynamics of endogenously-tagged PAR-3 during polarity establishment and maintenance phases.** Left: embryo from *par-1* heterozygote mother; Middle: Embryo from *par-1* homozygote mother; Right: *spd-5(RNAi)* embryo. All three embryos were imaged under identical conditions. Time is measured relative to the onset of maintenance phase. Time compression 90:1

Supplement 2 **Movie S2. Dynamics of FRAP recovery**. Embryo was subjected to photobleaching just before time 0. Time compression 18:1.

Supplement 3 **Movie S3. Fast imaging of endogenously-tagged PAR-3::GFP in an embryo depleted of myosin II heavy chain (*nmy-2(RNAi)*).** Time compression 1:1.

Supplement 4 **Movie S4. Single molecule imaging of endogenously-tagged PAR-3::GFP and transgenic ΔCR1 PAR-3::GFP.** (Left) endogenously-tagged PAR-3::GFP; (right) transgenic ΔCR1 PAR-3::GFP. Data were collected in streaming mode with 100% laser power at 15 frames per second to measure short-term mobilities and monomer dissociation rates. Time compression 2:1.

Supplement 5 **Movie S5. Single molecule observations of appearance events in control and *par-1(RNAi)* embryos expressing transgenic ΔCR1 PAR-3::GFP.** (left) Control; (right(par-1(RNAi). Data were collected with intermediate laser power in streaming mode at 20 frames per second. Time compression 1:1.

Supplement 6 **Movie S6. Distribution of endogenously-tagged PAR-3::GFP oligomers during maintenance phase in embryos from *par-1*/+ mothers subjected to the indicated RNAi depletions.** Time is measured relative to the onset of maintenance phase. Time compression: 90:1

Supplement 7 **Movie S7. Distribution of endogenously-tagged PAR-3::GFP oligomers during maintenance phase in embryos from *par-1*/*par-1* mothers subjected to the indicated RNAi depletions.** Time is measured relative to the onset of maintenance phase. Time compression: 90:1

1 **Supplementary Figure S1 (related to Figure 2) PAR-3 oligomer dynamics during maintenance phase in wild-type and *spd-5(RNAi)* embryos. (A)** Individual near-TIRF images from a time-lapse sequence showing PAR-3 oligomer intensities and distributions at the cell surface in control (*par-1* heterozygote) embryos at early establishment, late establishment and late maintenance phase. **(B-D)** Results of particle detection analysis showing **(B)** mean oligomer size, **(C)** oligomer density, and **(D)** total PAR-3 fluorescence density over time on the anterior (magenta) and posterior (blue) cortex in control embryos. Data are from n = 8 embryos aligned with respect to the onset of maintenance phase. The grey shaded area indicates maintenance phase. Solid lines indicate the mean and dots indicate SEM. at each time point. **(E)** Individual near-TIRF images from a time-lapse sequence showing PAR-3 oligomer intensities and distributions at the cell surface in *spd-5(RNAi)* embryos at early establishment, late establishment, and late maintenance phase. **(F-H)** Results of particle detection analysis showing **(F)** mean oligomer size, **(G)** oligomer density, and **(H)** total PAR-3 fluorescence density over time on the cortex. Data are from n = 6 embryos aligned with respect to the onset of maintenance phase. The grey shaded area indicates maintenance phase. Solid lines indicate the mean and dots indicate SEM. at each time point.
**Supplementary Figure S2 (related to Figure 4 and 6). Single molecule measurements of PAR-3::GFP and PAR-3(CR1)::GFP. (A-B)** Plots of RMSD distributions and release curves for single molecules of PAR-3::GFP in *par-1* heterozygotes. **(A)** Distributions of root mean square displacements after t = 200 msec (RMSD_200_) measured for molecules tracked on the anterior and posterior cortex in n = 8 embryos. Each column represents data obtained using a different integrated intensity threshold for detecting single molecules, as indicated by the value for intensity threshold. Dark curve indicates simulated RMSD_200_ distributions for molecules undergoing Brownian diffusion with diffusivity = 0.1 μm^2^/sec. **(B)** Release curves measured for single molecules of PAR-3::GFP on the anterior and posterior cortex of wild type embryos under continuous illumination fort different feature detection thresholds. Thin traces show release curves for individual embryos (n = 8); Thick traces show data summed for all embryos. Data were normalized by total numbers of anterior tracks. **(C-D)** Plots of RMSD distributions and release curves for single molecules of PAR-3(ΔCR1)::GFP in *par-1* heterozygotes. **(C)** Distributions of root mean square displacements after t = 200 msec (RMSD_200_) measured for molecules tracked on the anterior and posterior cortex in n = 9 embryos. Each column represents data obtained using a different integrated intensity threshold for detecting single molecules, as indicated by the value for feasturre detection threshold. Dark curve indicates simulated RMSD_200_ distributions for molecules undergoing Brownian diffusion with diffusivity = 0.1 μm^2^/sec. **(D)** Release curves measured for single molecules of PAR-3::GFP on the anterior and posterior cortex of wild type embryos under continuous illumination fort different feature detection thresholds. Thin traces show release curves for individual embryos (n = 9); Thick traces show data summed for all embryos. Data were normalized by total numbers of anterior tracks.
**Supplementary Figure S3 (related to Figure 5). Examples of different phenotype classes in the PAR-3 depletion experiment shown in Figure 5D–E.** Top and bottom micrographs show the distribution of PAR-3 at early and late maintenance respectively. We observed three distinct classes: (left) polarity established and maintained, (middle) polarity established and not maintained and (right) polarity never established. For the middle class, the kymograph confirms that the loss of PAR-3 asymmetry was not due to redistribution of PAR-3 by posterior-directed cortical flows.
**Supplementary Figure S4 (related to Figure 8). Mobilities and release curves for single molecules of PAR-3::GFP in control, *cdc-42(RNAi)* and *par-6(RNAi)* embryos. (A)** Release curves for single molecules of transgenic PAR-3::GFP measured in control, *cdc-42(RNAi)* and *par-6(RNAi)* embryos during maintenance phase using high laser power, short exposures and low duty ratios to achieve accurate tracking with low photobleaching. All data were collected under identical conditions. Each thin curve represents data from a single embryo. Thick curves represent the average over (control: n = 8), (*cdc-42(RNAi)*: n = 12) and (*par-6(RNAi)*: n = 14) embryos. (**B-C**) Distributions of root mean square displacements after 200 msec (RMSD_200) and release curves for anterior (orange) and posterior (green) single molecules measured in (**B**) control and (**C**) *par-6(RNAi); mrck-1(RNAi)* embryos measured in early maintenance phase. (**D-E**) Distributions of root mean square displacements after 200 msec (RMSD_200) and release curves for anterior (orange) and posterior (green) single molecules measured in (**D**) control and (**E**) *cdc-42(RNAi)* embryos measured in late maintenance phase.

## Figures and Tables

**Table 1.** *C. elegans* strains used in this study.

Name	Genotype	Origin
Em267	par-3(it298[PAR-3::GFP]) III	Kenneth Kemphues
Em292	par-3(it298[PAR-3::GFP]) III; rol-4(sc8) par-1(it0051) V/DnT1 IV;V	Kenneth Kemphues
Em307	itIs268[ppar-3::PAR-3(V80D D198K)::GFP + unc-119(+)]; unc-119(ed3) III #5	Kenneth Kemphues
Em327	*par-6(cp60[par-6::mKate2::3xMyc + LoxPunc-119(+) LoxP]) I par-3(cp54[mNG::3xFlag::par-3]) III;*	Daniel Dickinson
