# Supplementary material for: Oligomerization and positive feedback on membrane recruitment encode dynamically stable PAR-3 asymmetries in the C. elegans zygote
Source: bioRxiv. 2025 Aug 25:2023.08.04.552031. Preprint. [Version 3] doi: 10.1101/2023.08.04.552031 (PMC11383301; doi:10.1101/2023.08.04.552031)

bioRxiv preprint doi: https://doi.org/10.1101/2023.08.04.552031; this version posted August 25, 2025. The copyright holder for this preprint (which was not certified by peer review) is the author/funder, who has granted bioRxiv a license to display the preprint in perpetuity. It is made available under aCC-BY 4.0 International license.

Supplementary Figure S1 (related to Figure 2) PAR-3 oligomer dynamics during maintenance phase in *spd-5(RNAi)* embryos. (A) Individual near-TIRF images from a time-lapse sequence showing PAR-3 oligomer intensities and distributions at the cell surface in *spd-5(RNAi)* embryos at early establishment, late establishment, and late maintenance phase. **(B-D)** Results of particle detection analysis showing **(B)** mean oligomer size, **(C)** oligomer density, and **(D)** total PAR-3 fluorescence density over time on the cortex. Data are from n = 6 embryos aligned with respect to the onset of maintenance phase. The grey shaded area indicates maintenance phase. Solid lines indicate the mean and dots indicate SEM at each time point.

Supplementary Figure S2 (related to Figure 3 and 5). Single molecule measurements of size-dependent recruitment and dissociation of PAR-3::GFP oligomers. (A) Overlay of the distributions of background-subtracted intensities of (blue) newly detected PAR-3::GFP speckles from the data in (A) and (peach) single molecule speckles after photobleaching in the same cells. **(B)** Estimated rate constants for dissociation rates of PAR-3::GFP oligomers from the membrane

bioRxiv preprint doi: https://doi.org/10.1101/2023.08.04.552031; this version posted August 25, 2025. The copyright holder for this preprint (which was not certified by peer review) is the author/funder, who has granted bioRxiv a license to display the preprint in perpetuity. It is made available under aCC-BY 4.0 International license.

as a function of inferred number of subunits n, measured as described for mNG::PAR-3 in Figure 3K. Error bars represent the SEM. Data were compiled from n = 6 individual embryos.

Supplementary Figure S3 (related to Figure 4). Measured PAR-3 release rates and mobilities do not depend sensitively on single molecule detection threshold. (A) Distributions of single molecule displacements after 100 msec (D_100) measured for endogenously-tagged PAR-3::GFP molecules tracked on the anterior and posterior cortex in n = 9 embryos. Each column represents data obtained using a different integrated intensity threshold for detecting single molecules. Solid curves show least squares best fit to weighted D_100 distributions measured for oligomers and for simulated Brownian diffusion with D = 0.1 μm^2/sec, as in Figure 4.. **(B)** Release curves measured for single molecules of PAR-3::GFP on the anterior and posterior cortex of wild type embryos under continuous illumination for different intensity thresholds. Thin traces show release curves for individual embryos (n = 9); Thick traces show pooled data for all embryos. Data were normalized by total numbers of anterior tracks. **(C)** Same data plotted in **(B)**, but with anterior and posterior data individually normalized to emphasize differences in release kinetics. **(D)** Release curves measured for posterior single molecules of oligomerization-defective PAR-3. Dashed lines indicate data fit to a weighted sum of two exponentials. **(E)** Corresponding estimate of effective k_{off} from the data in (D). n = 11 embryos. Error bars represent 95% confidence intervals obtained by bootstrap sampling.

Supplementary Figure S4 (related to Figure 5). Single molecule analysis of monomer recruitment and release rate asymmetries for a second form of oligomerization-defective PAR-3 (PAR-3$^{CR1\Delta(69-82)}$::GFP) (A) Representative images and distribution of single molecule

bioRxiv preprint doi: https://doi.org/10.1101/2023.08.04.552031; this version posted August 25, 2025. The copyright holder for this preprint (which was not certified by peer review) is the author/funder, who has granted bioRxiv a license to display the preprint in perpetuity. It is made available under aCC-BY 4.0 International license.

appearance events for oligomerization-defective PAR-3$^{CR1\Delta(69\text{-}82)}$::GFP, detected by single molecule imaging. Each blue dot represents a single binding event. **(B)** Release curves for (n = 10) embryos. Faint lines show data for individual embryos, thick lines for pooled data, and dashed lines indicate the exponential fits to pooled data. Inset shows the extrapolated zero-crossings from which the total numbers of binding events were inferred. Anterior and posterior data were normalized to total numbers of anterior binding events to highlight the recruitment asymmetry. **(C)** A:P recruitment ratios measured from the data in **(B)**. Error bars indicate 95% confidence intervals obtained from bootstrap sampling. **(D)** Pooled release curves and exponential fits with anterior and posterior data separately normalized to highlight differences in release rates. **(E)** A:P ratios of monomer release rates for fast and slow components (both) and for fast components alone (fast). Error bars indicate 95% confidence intervals obtained from bootstrap sampling. **(F)** Distributions of short-term displacements (D_100) for anterior and posterior molecules, measured over all trajectories (top row), long trajectories (middle row; duration > 2 sec), and short trajectories (bottom row; duration = 250 msec). Solid curves show data fits to weighted sums of D_100 distributions for oligomers and for simulated Brownian diffusion with diffusivity D = 0.1 μm^2/sec (as in Fig 4C).

Supplementary Figure S5 (related to Figure 6). Further details underlying the model and its comparison to data. (A) (left) Release curves for PAR-3^{V80D,D138K}::GFP molecules measured in the same embryos during early and late maintenance phase. Anterior and posterior data were normalized to total numbers of anterior binding events to highlight the recruitment asymmetry. (right) A:P recruitment ratios measured from the same data (n = 10 embryos). Error bars indicate 95% confidence intervals obtained from bootstrap sampling. **(B)** Kymographs illustrating patterns

bioRxiv preprint doi: https://doi.org/10.1101/2023.08.04.552031; this version posted August 25, 2025. The copyright holder for this preprint (which was not certified by peer review) is the author/funder, who has granted bioRxiv a license to display the preprint in perpetuity. It is made available under aCC-BY 4.0 International license.

of cortical flow during establishment phase in control embryos, *par-3* mutant embryos, and *par-3* mutant embryos depleted of PAR-1 by RNAi. **(C)** Examples of different phenotype classes in the PAR-3 depletion experiment shown in Figure 5D. Top and bottom micrographs show the distribution of PAR-3 at early and late maintenance respectively. We observed three distinct classes: (left) polarity established and maintained, (middle) polarity established and not maintained and (right) polarity never established. For the middle class, the kymograph confirms that the loss of PAR-3 asymmetry was not due to redistribution of PAR-3 by posterior-directed cortical flows.

Supplementary Figure S6 (related to Figure 8). Mobilities and release curves for single molecules of oligomerization-defective PAR-3^{V80D,D138K}::GFP under different perturbations. **(A,E,I,M)** Release curves for PAR-3^{V80D,D138K}::GFP molecules in: **(A)** control (n = 9) and *par-6(RNAi);mrck-1(RNAi)* (n = 12) embryos, **(E)** *pkc-3as* embryos treated with DMSO (n = 6) or 50 µM 1-NA-PP1 (n = 8), **(I)** *par-3* mutant embryos partially rescued by transgenic PAR-3^{V80D,D138K}::GFP (n = 6), and **(M)** control (n = 6) and *cdc-42 (RNAi)* (n = 6) embryos. Faint lines show data for individual embryos, thick lines for pooled data, and dashed lines indicate the exponential fits to pooled data. Anterior and posterior data were normalized by total numbers of anterior binding events to highlight the recruitment asymmetry. **(B,F,J,N).** The same data with anterior and posterior release curves normalized using total numbers of anterior and posterior binding events respectively. **(C,G,K,O)**. Distributions of short-term displacements (D_100) for the indicated genotypes and perturbations. Solid curves show data fits to weighted sums of D_100 distributions for oligomers and for simulated Brownian diffusion with diffusivity D = 0.1 µm^2/sec (as in Fig 4C). Recruitment ratios computed from these data are shown in Figure 8. **(D,H,L,P)**. Dissociation ratios measured for the indicated genotypes and perturbations.

bioRxiv preprint doi: https://doi.org/10.1101/2023.08.04.552031; this version posted August 25, 2025. The copyright holder for this preprint (which was not certified by peer review) is the author/funder, who has granted bioRxiv a license to display the preprint in perpetuity. It is made available under aCC-BY 4.0 International license.

(both): ratio of effective release rates for slow and fast dissociating monomers. **(fast):** Estimated ratio of release rates for fast-dissociating monomer fraction. **(slow):** Estimated ratio of release rates for slow-dissociating monomer fraction. Error bars represent 95% confidence intervals obtained by bootstrap sampling.

Supplemental Movie Legends

Movie S1. Dynamics of endogenously-tagged PAR-3 during polarity establishment and maintenance phases. Left: embryo from *par-1* heterozygote mother; Middle: Embryo from *par-1* homozygote mother; Right: *spd-5(RNAi)* embryo. All three embryos were imaged under identical conditions. Time is measured relative to the onset of maintenance phase. Time compression 90:1

Movie S2. Dynamics of FRAP recovery. Embryo was subjected to photobleaching just before time 0. Time compression 18:1.

Movie S3. Fast imaging of endogenously-tagged mNeonGreen::PAR-3 in an embryo depleted of myosin II heavy chain (*nmy-2(RNAi)*). Time compression 1:1.

Movie S4. Single molecule imaging of endogenously-tagged PAR-3::GFP. Data were collected with high laser power in streaming mode at 20 frames per second to measure short-term mobilities and monomer dissociation rates. Time compression 1.5:1.

bioRxiv preprint doi: https://doi.org/10.1101/2023.08.04.552031; this version posted August 25, 2025. The copyright holder for this preprint (which was not certified by peer review) is the author/funder, who has granted bioRxiv a license to display the preprint in perpetuity. It is made available under aCC-BY 4.0 International license.

Movie S5. Single molecule observations of appearance events in control and *par-1(RNAi)* embryos expressing transgenic PAR-3::GFP. (left) *control*; (right) *par-1(RNAi)*. Data were collected with high laser power in streaming mode at 20 frames per second to measure monomer recruitment and dissociation kinetics and short-term mobilities. Time compression 1.5:1.

Movie S6. Distribution of endogenously-tagged PAR-3::GFP oligomers during maintenance phase in embryos from *par-1*/+ mothers subjected to the indicated RNAi depletions. Time is measured relative to the onset of maintenance phase. Time compression: 90:1

Movie S7. Distribution of endogenously-tagged PAR-3::GFP oligomers during maintenance phase in embryos from *par-1*/*par-1* mothers subjected to the indicated RNAi depletions. Time is measured relative to the onset of maintenance phase. Time compression: 90:1

bioRxiv preprint doi: https://doi.org/10.1101/2023.08.04.552031; this version posted August 25, 2025. The copyright holder for this preprint (which was not certified by peer review) is the author/funder, who has granted bioRxiv a license to display the preprint in perpetuity. It is made available under aCC-BY 4.0 International license.

Lang et al, Figure S1

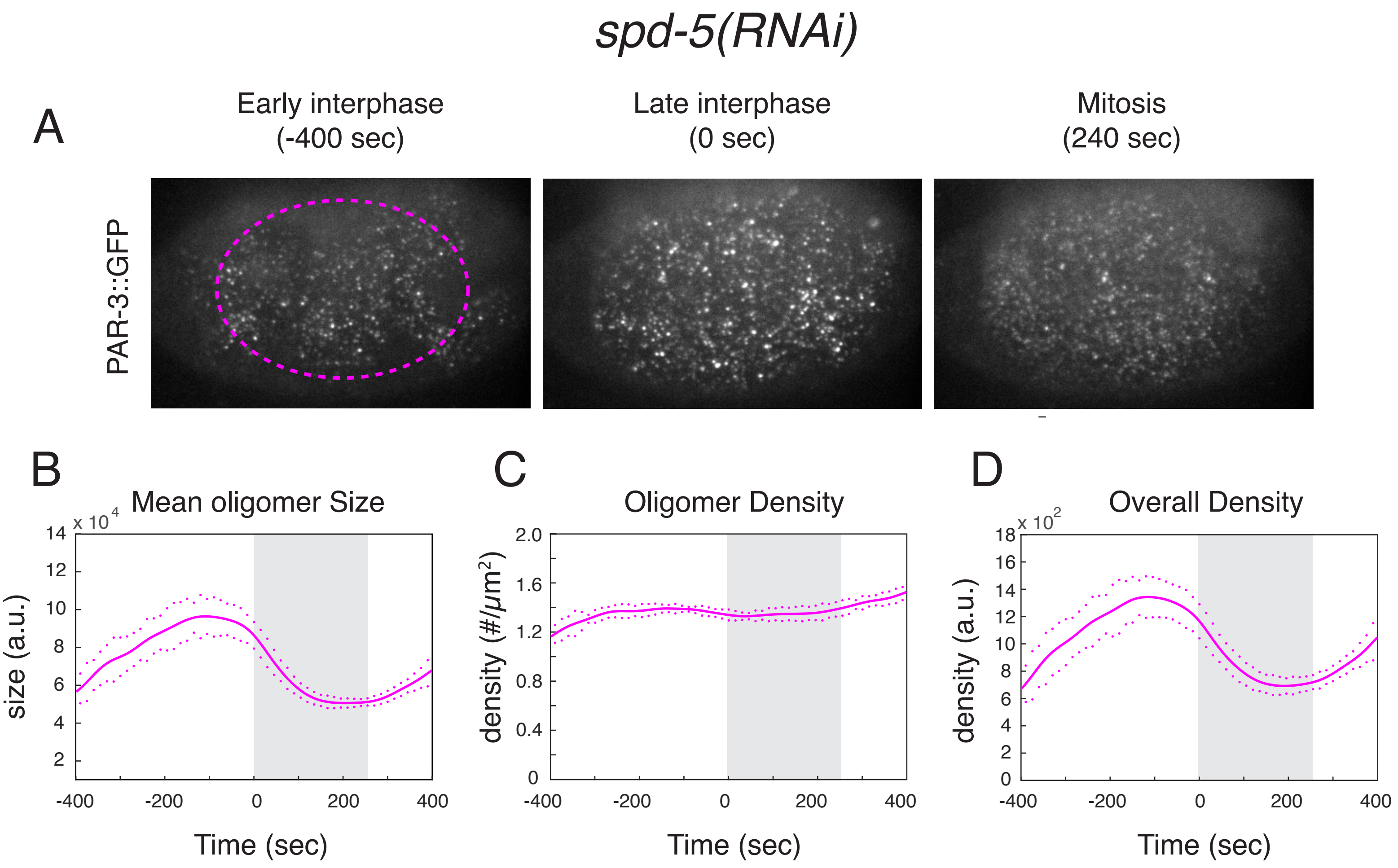

bioRxiv preprint doi: https://doi.org/10.1101/2023.08.04.552031; this version posted August 25, 2025. The copyright holder for this preprint (which was not certified by peer review) is the author/funder, who has granted bioRxiv a license to display the preprint in perpetuity. It is made available under aCC-BY 4.0 International license.

Lang et al, Figure S2

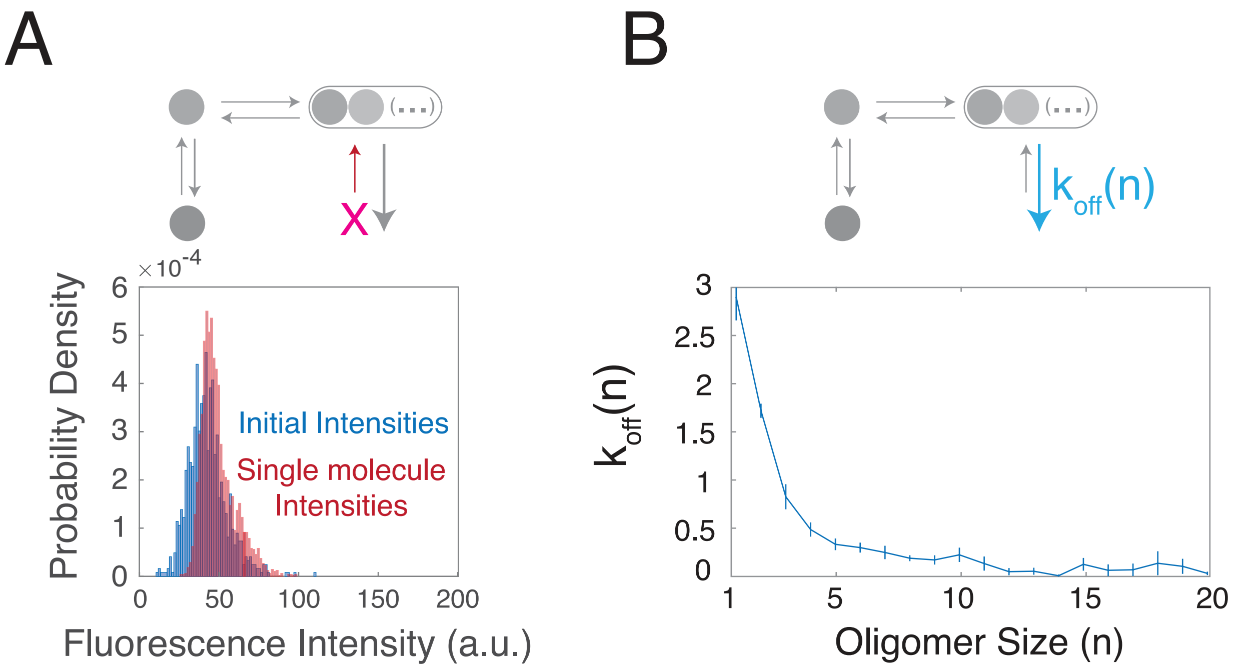

bioRxiv preprint doi: https://doi.org/10.1101/2023.08.04.552031; this version posted August 25, 2025. The copyright holder for this preprint (which was not certified by peer review) is the author/funder, who has granted bioRxiv a license to display the preprint in perpetuity. It is made available under aCC-BY 4.0 International license.

Lang et al, Figure S3

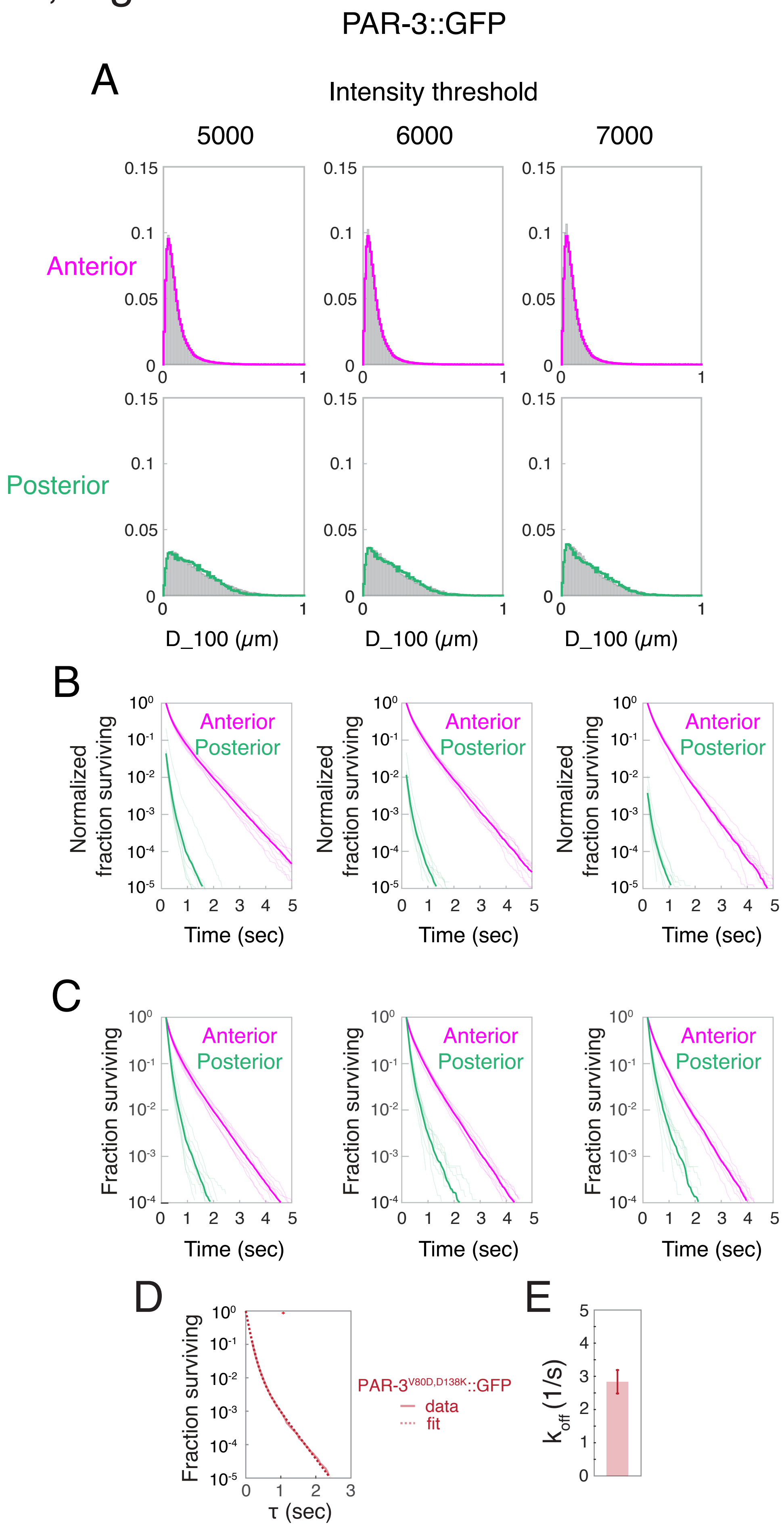

bioRxiv preprint doi: https://doi.org/10.1101/2023.08.04.552031; this version posted August 25, 2025. The copyright holder for this preprint (which was not certified by peer review) is the author/funder, who has granted bioRxiv a license to display the preprint in perpetuity. It is made available under aCC-BY 4.0 International license.

Lang et al, Figure S4

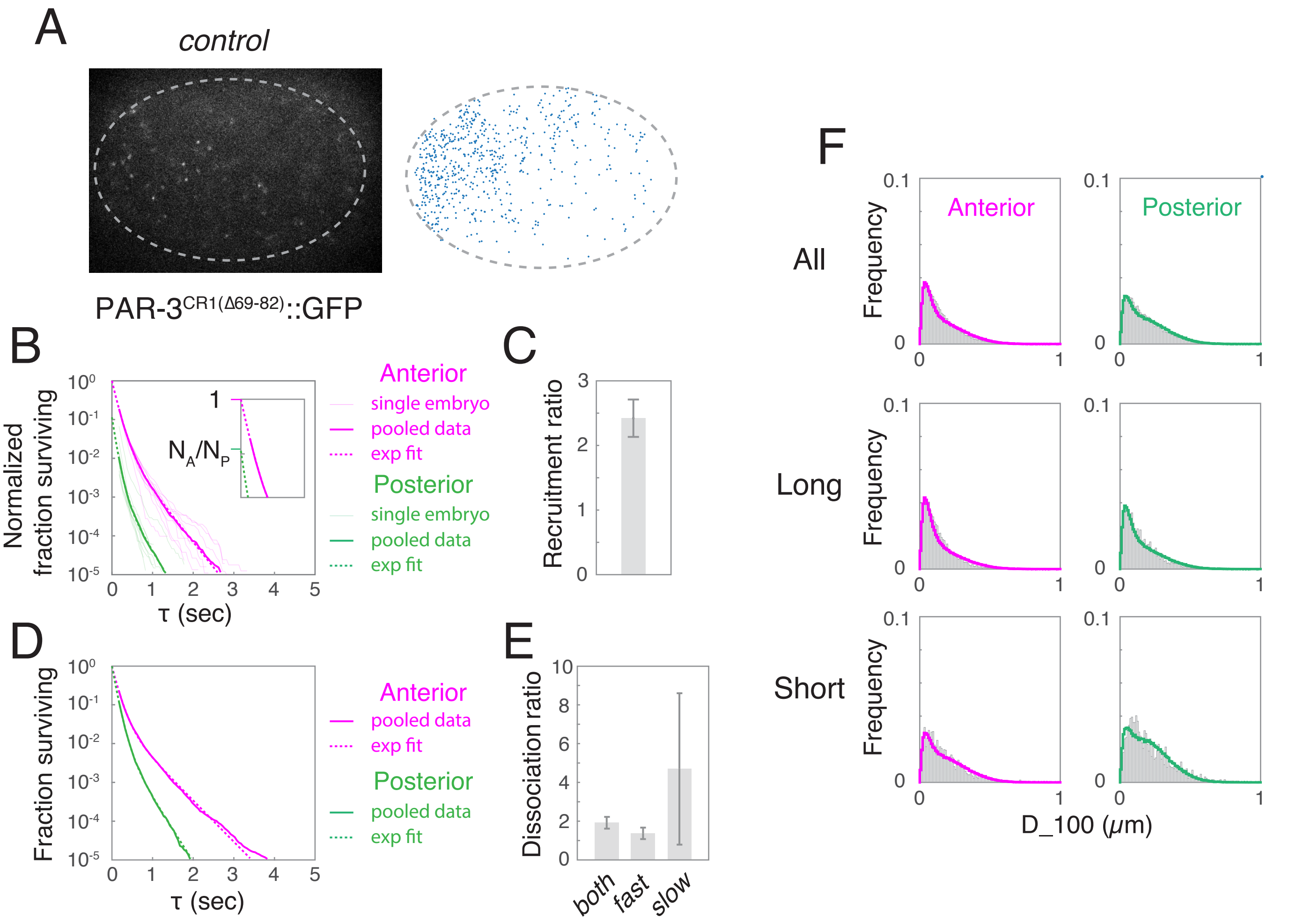

bioRxiv preprint doi: https://doi.org/10.1101/2023.08.04.552031; this version posted August 25, 2025. The copyright holder for this preprint (which was not certified by peer review) is the author/funder, who has granted bioRxiv a license to display the preprint in perpetuity. It is made available under aCC-BY 4.0 International license.

Lang et al, Figure S5

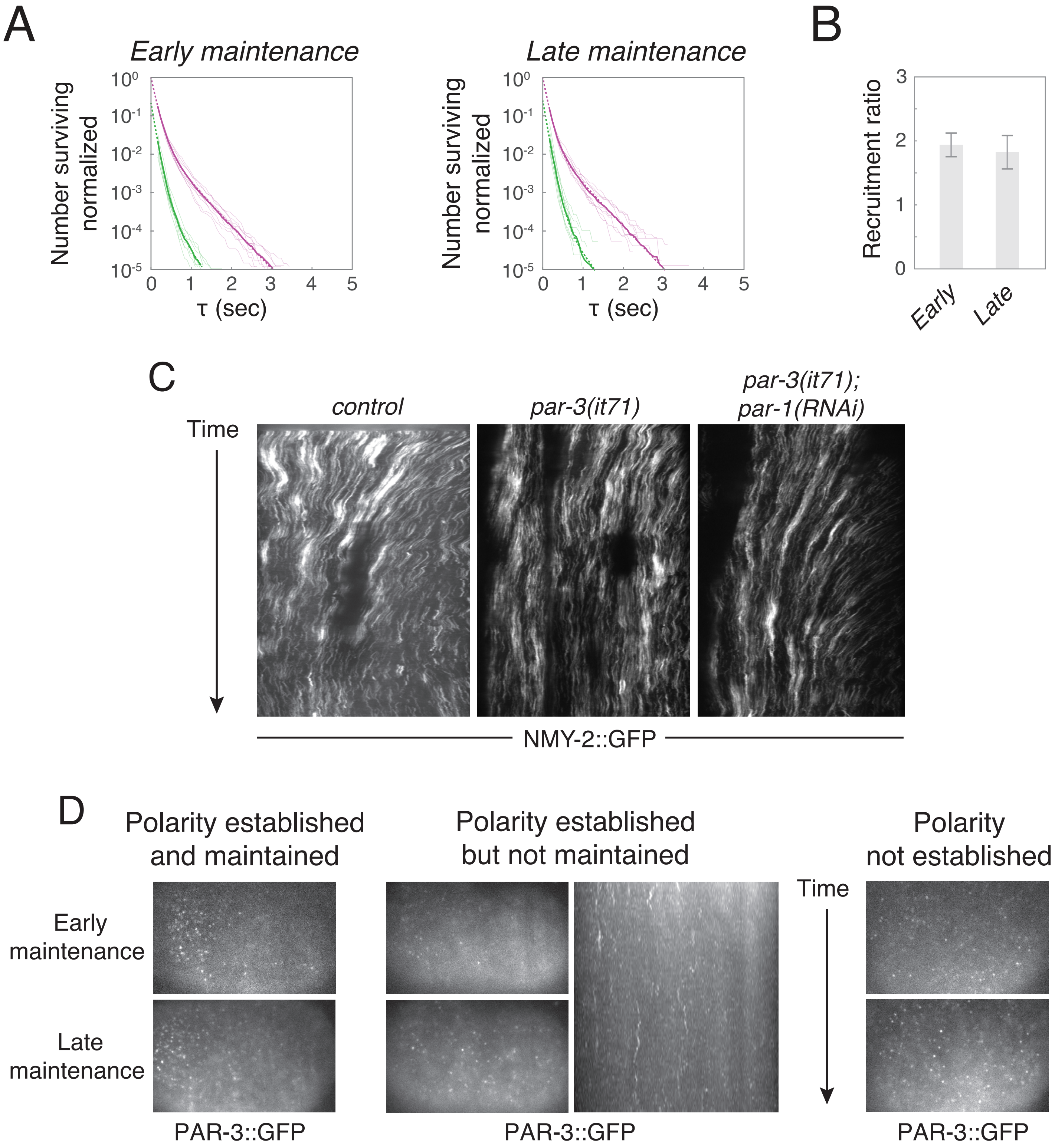

bioRxiv preprint doi: https://doi.org/10.1101/2023.08.04.552031; this version posted August 25, 2025. The copyright holder for this preprint (which was not certified by peer review) is the author/funder, who has granted bioRxiv a license to display the preprint in perpetuity. It is made available under aCC-BY 4.0 International license.

Lang et al, Figure S6

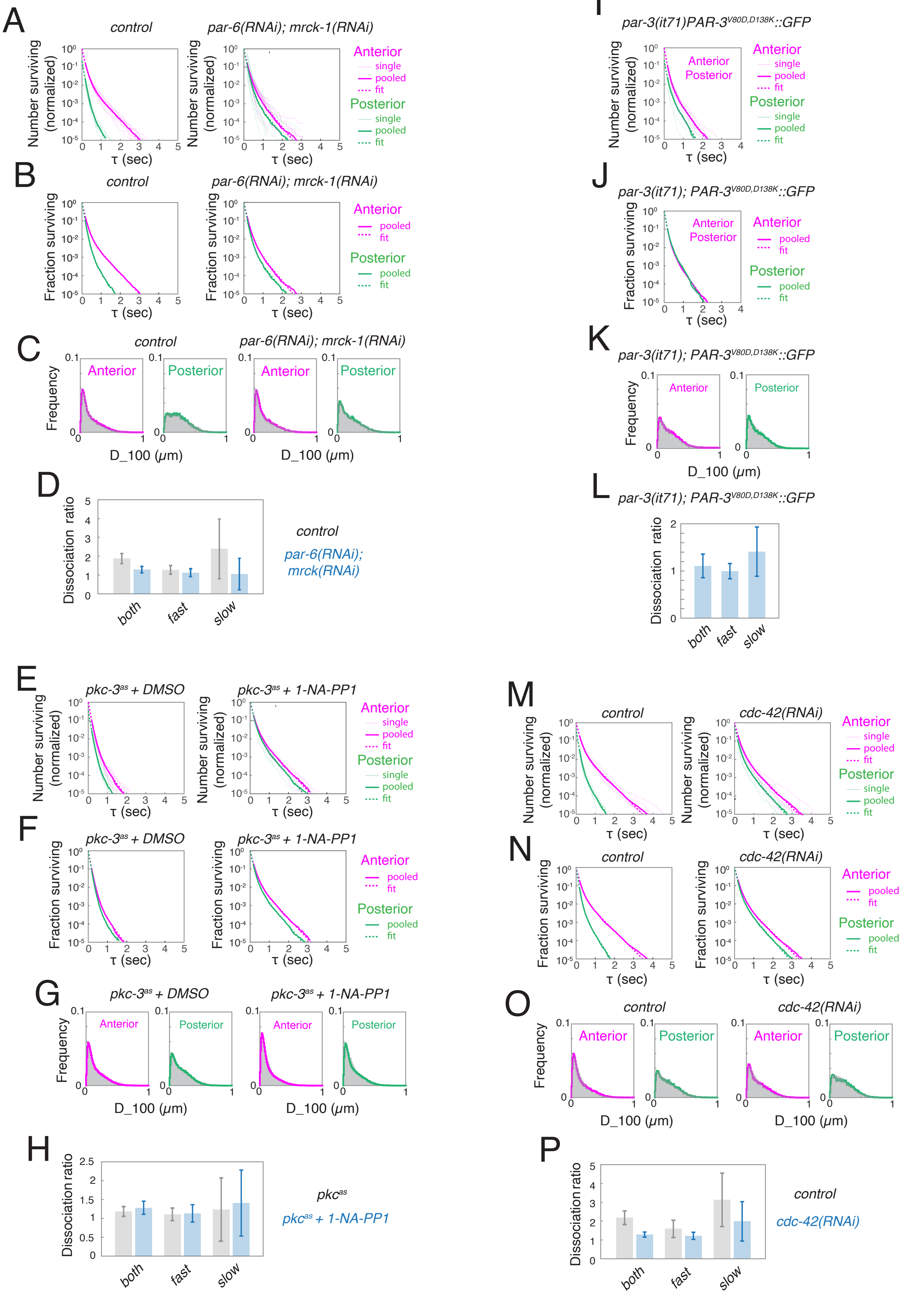

Supplement: Supplement 9 [file NIHPP2023.08.04.552031v3-supplement-9.pdf]
