# Supplementary material for: Oligomerization and positive feedback on membrane recruitment encode dynamically stable PAR-3 asymmetries in the C. elegans zygote
Source: bioRxiv. 2024 Aug 28:2023.08.04.552031. Preprint. [Version 2] doi: 10.1101/2023.08.04.552031 (PMC11383301; doi:10.1101/2023.08.04.552031)
Supplement: Supplementary file 1 [file NIHPP2023.08.04.552031v2-supplement-1.pdf]

Figure S1 bioRxiv preprint doi: https://doi.org/10.1101/2023.08.04.552031; this version posted August 28, 2024. The copyright holder for this preprint (which was not certified by peer review) is the author/funder, who has granted bioRxiv a license to display the preprint in perpetuity. It is made available under aCC-BY 4.0 International license.

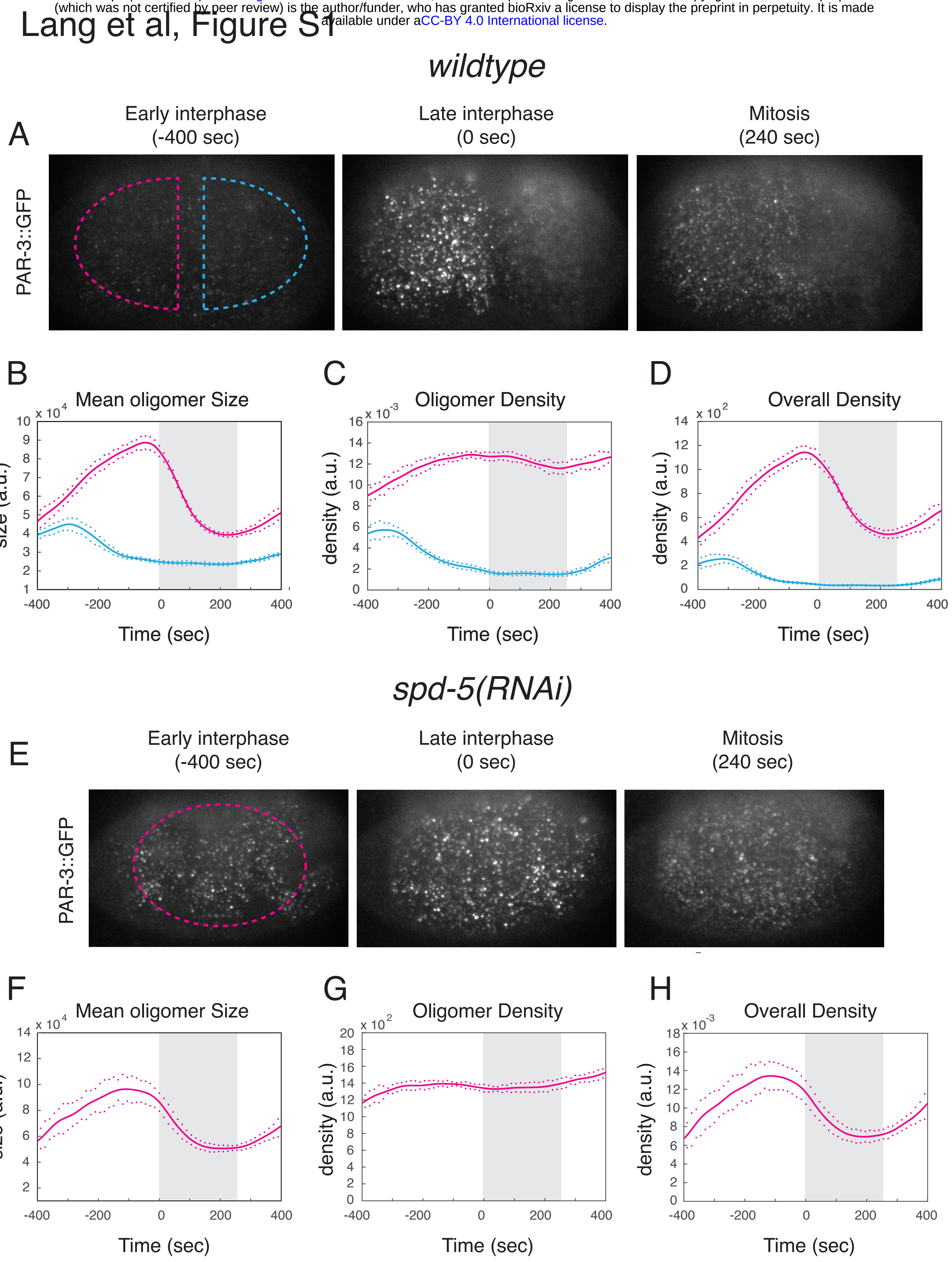

Figure S2

bioRxiv preprint doi: https://doi.org/10.1101/2023.08.04.552031; this version posted August 28, 2024. The copyright holder for this preprint (which was not certified by peer review) is the author/funder, who has granted bioRxiv a license to display the preprint in perpetuity. It is made available under aCC-BY 4.0 International license.

Lang et al, Figure S2

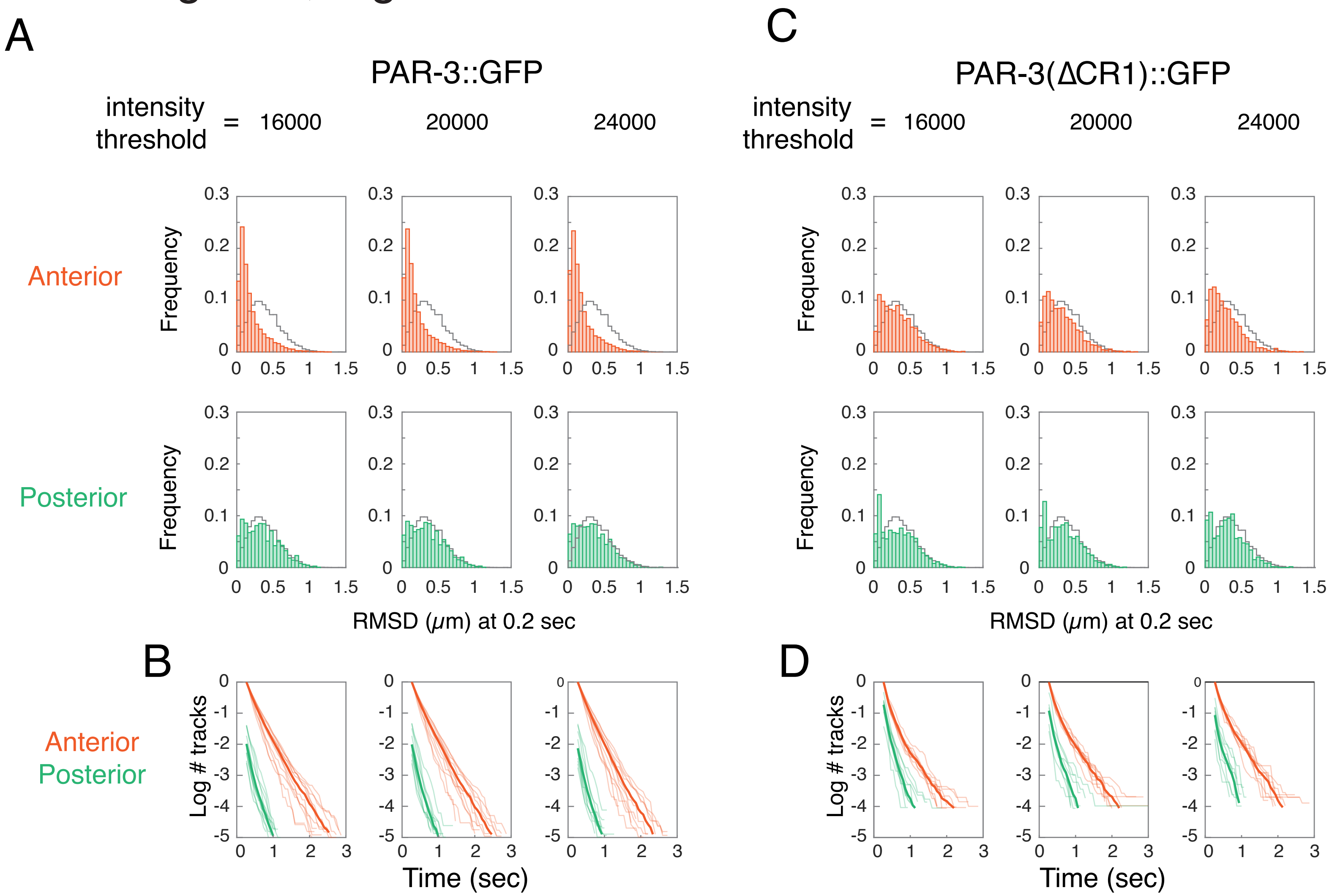

bioRxiv preprint doi: https://doi.org/10.1101/2023.08.04.552031; this version posted August 28, 2024. The copyright holder for this preprint (which was not certified by peer review) is the author/funder, who has granted bioRxiv a license to display the preprint in perpetuity. It is made available under aCC-BY 4.0 International license.

Lang et al, Figure S3

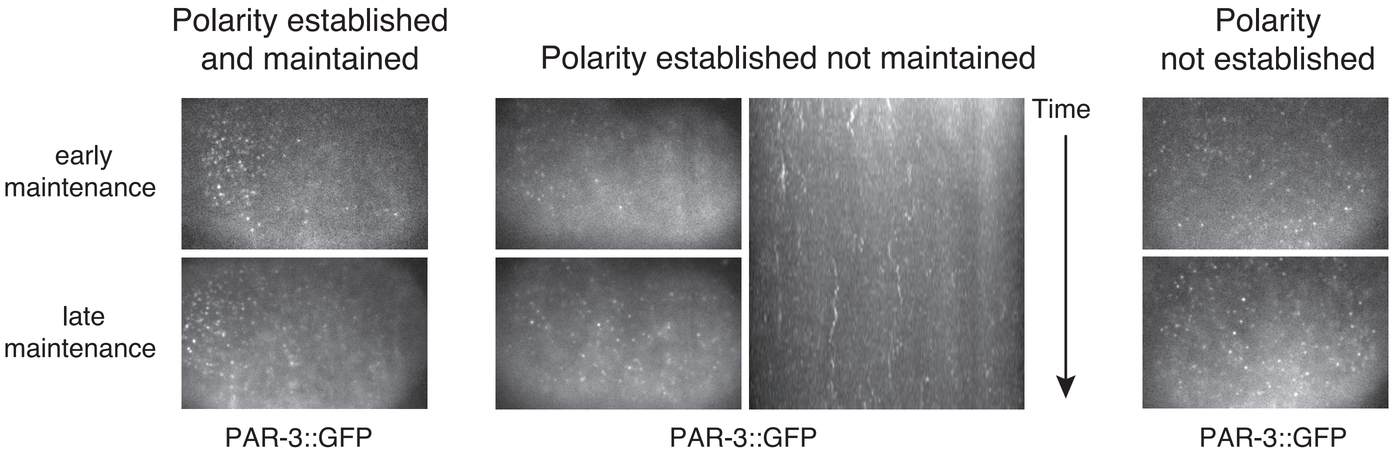

Figure S4

bioRxiv preprint doi: https://doi.org/10.1101/2023.08.04.552031; this version posted August 28, 2024. The copyright holder for this preprint (which was not certified by peer review) is the author/funder, who has granted bioRxiv a license to display the preprint in perpetuity. It is made available under aCC-BY 4.0 International license.

Lang et al. Figure S4

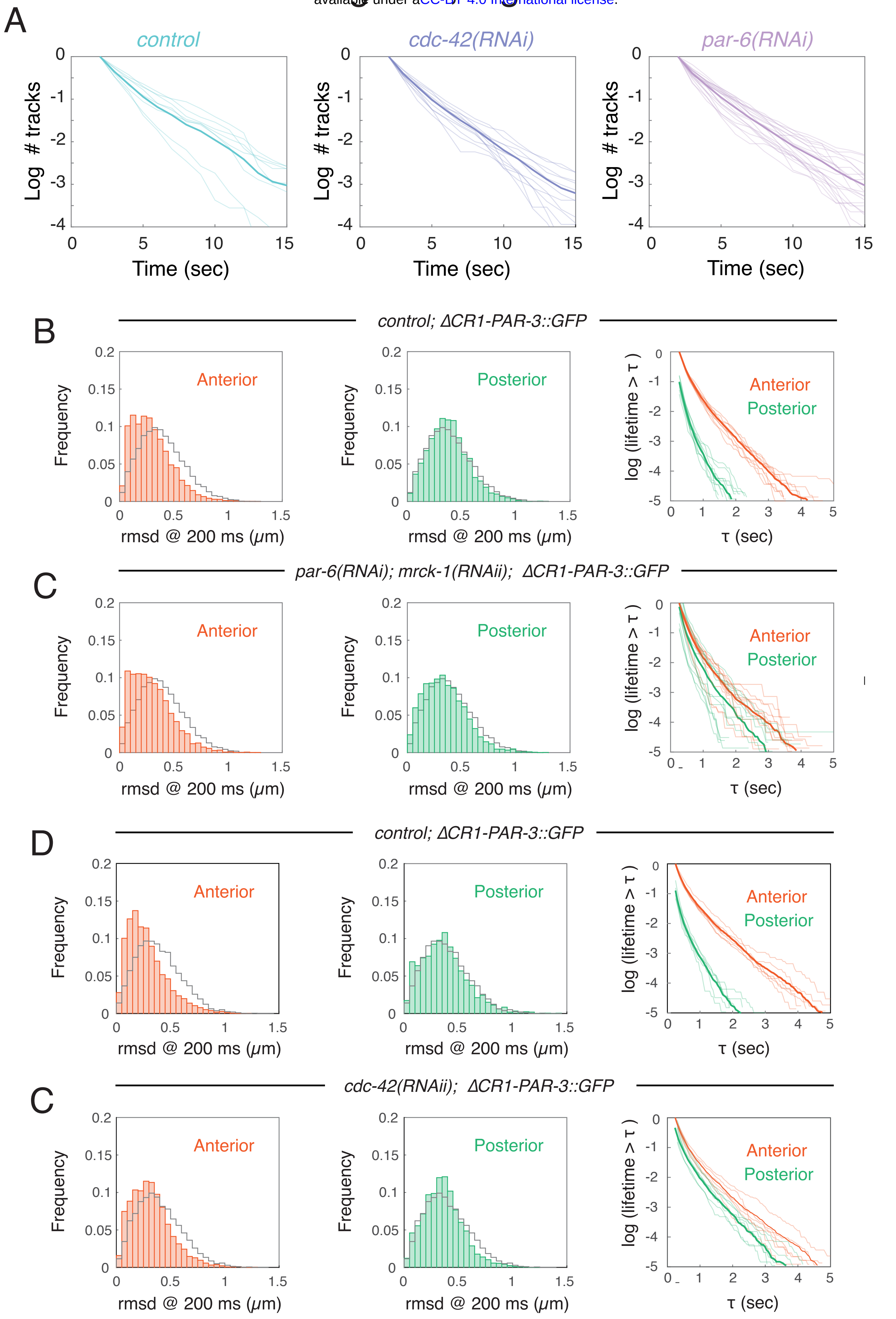

Figure S5

bioRxiv preprint doi: https://doi.org/10.1101/2023.08.04.552031; this version posted August 28, 2024. The copyright holder for this preprint (which was not certified by peer review) is the author/funder, who has granted bioRxiv a license to display the preprint in perpetuity. It is made available under aCC-BY 4.0 International license.

Lang et al. Figure S5

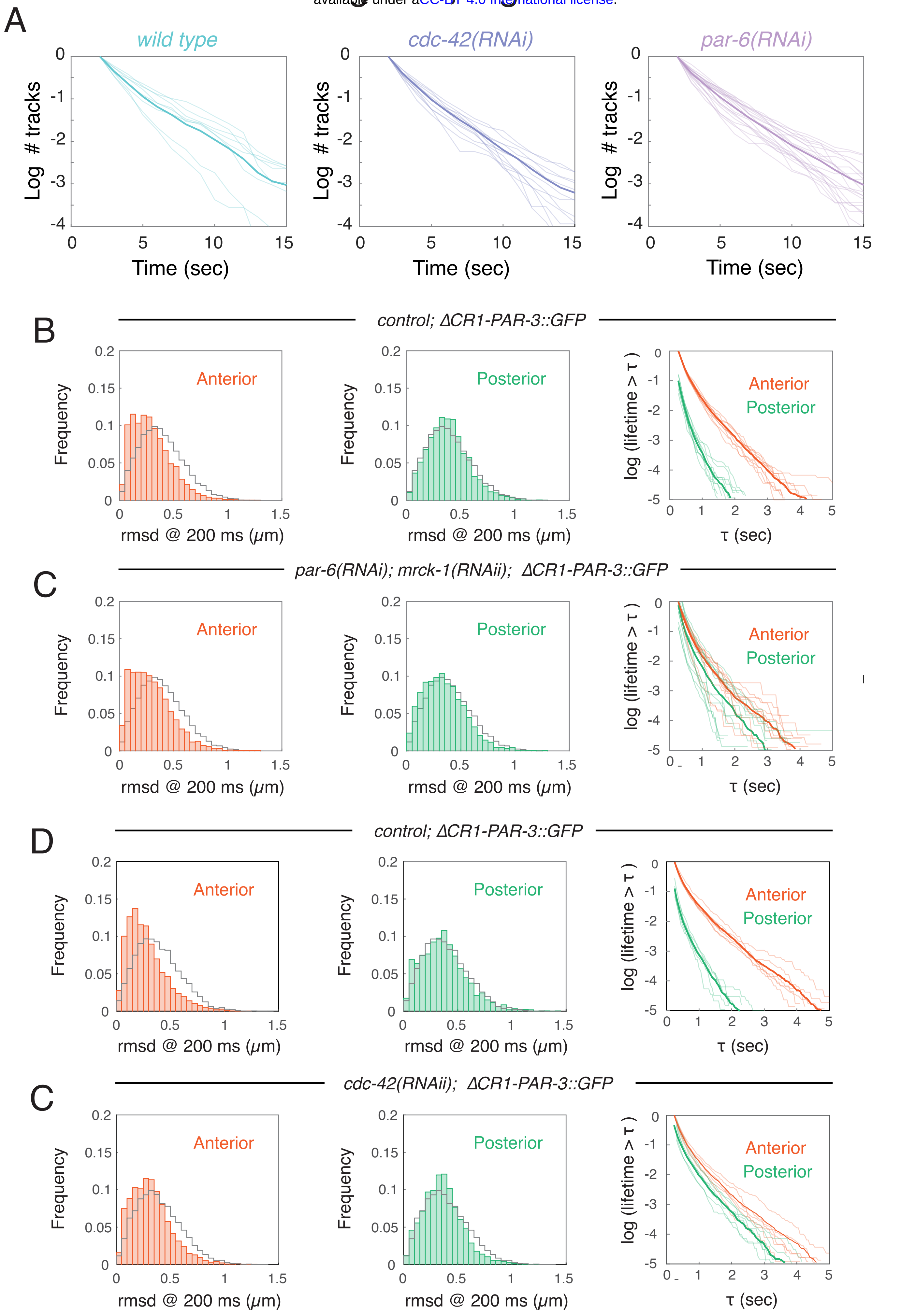

bioRxiv preprint doi: https://doi.org/10.1101/2023.08.04.552031; this version posted August 28, 2024. The copyright holder for this preprint (which was not certified by peer review) is the author/funder, who has granted bioRxiv a license to display the preprint in perpetuity. It is made available under aCC-BY 4.0 International license.

Mathematical appendix: Oligomerization and positive feedback on membrane recruitment encode dynamically stable PAR-3 asymmetries in the C. elegans zygote

Charles F. Lang, Ondrej Maxian, Alexander Anneken, and Edwin M. Munro

In this section we describe the derivation, parameterization and analysis of a mathematical model for PAR-3 kinetics. The basic approach follows our previous theoretical analysis of oligomerization by peripheral membrane binding proteins [6]. Briefly, as schematized in Figs. 3A and 7A, we assume a well-mixed cytoplasmic pool of PAR-3 monomers which bind reversibly to the plasma membrane. At the membrane, monomers assemble reversibly into linear oligomers with size-dependent mobilities and dissociation rates. Finally, we assume that membrane-bound PAR-3 feeds back indirectly to promote the attachment of PAR-3 monomers to the membrane. Letting $A_n(x)$ be the density (number/length) of membrane-bound oligomers of size n at point x, and A_c be the concentration of cytoplasmic PAR-3, the density of membrane-bound oligomers evolves according to the following equations [6, Eq. (1)]

$$\partial_t A_1 = D_1 \partial_x^2 A_1 + \left(k_{\text{basal}} + k_f f(A)\right) A_{\text{c}} - k_{\text{off}} A_1 \qquad \text{(M1a)}$$

$$+ 2k_{\text{dep}} A_2 - 2k_{\text{pol}} A_1^2 + \sum_{n=3}^{N} \left(k_{\text{dep}} A_n - k_{\text{pol}} A_1 A_{n-1}\right)$$

$$\partial_t A_n = k_{\text{pol}} A_1 \left(A_{n-1} - A_n\right) - k_{\text{dep}} \left(A_n - A_{n+1}\right) \qquad N > n \geq 2 \qquad \text{(M1b)}$$

$$\partial_t A_N = k_{\text{pol}} A_1 A_{N-1} - k_{\text{dep}} A_N \qquad \text{(M1c)}$$

$$A_{\text{c}} = \frac{1}{hL} \left(A^{(\text{Tot})} L - \int_0^L A(x)\, dx \right) \qquad A(x) = \sum_{n=1}^{N} n A_n(x). \qquad \text{(M1d)}$$

The first line of (M1a) describes the membrane attachment and detachment of PAR-3 monomers, with basal rates governed by k_{basal} and k_{off}, and density-dependent feedback on monomer attachment governed by $k_f f(A)$, whose form we consider below. The second line of (M1a), combined

bioRxiv preprint doi: https://doi.org/10.1101/2023.08.04.552031; this version posted August 28, 2024. The copyright holder for this preprint (which was not certified by peer review) is the author/funder, who has granted bioRxiv a license to display the preprint in perpetuity. It is made available under aCC-BY 4.0 International license.

with (M1b) and (M1c), describes the dynamics of polymerization on the membrane, with forward and reverse rate constants k_{pol} and k_{dep}. Finally, the cytoplasmic concentration A_{c} is determined in (M1d) from the conservation of total protein in the system, where $A(x)$ is the total bound density at each point on the membrane and $A^{(\text{Tot})}$ is the density when all PAR-3 is uniformly bound to the membrane.

The forms of equations (M1) reflect several important simplifying assumptions, which are justified by our experimental observations. First, our experimental data (Fig. 3K) show that oligomer mobilities and dissociation rates fall sharply with increasing oligomer size. Therefore we neglect both oligomer diffusion and detachment, as in [6]. Second, our experimental observations (see main text) suggest that direct attachment of cytoplasmic monomers to membrane-bound PAR-3 oligomers makes a relatively small contribution to the total flux of PAR-3 monomers onto the membrane. In previous work [6], we showed that direct attachment effectively reduces or increases the strength of indirect feedback, depending on the regime considered. Thus we do not explicitly model the direct attachment of cytoplasmic monomers to membrane-bound oligomers, instead assuming that the weak effects of this attachment are implicit in the feedback strength.

1 Nondimensionalization and parameter estimation

To estimate the model parameters and compare model predictions to experimental observations, we scale the equations by appropriate density ($A^{(\text{Tot,WT})}$, which represents $A^{(\text{Tot})}$ under wild type conditions), length (L), and time ($1/k_{\text{dep}}$) scales. Since polymerization dynamics are much slower than detachment, ($1/k_{\text{dep}}$) is the time a typical PAR-3 molecule spends on the membrane. Accordingly, we define the dimensionless (hatted) variables

$$x = \hat{x}L, \qquad t = \hat{t}/k_{\text{dep}}, \qquad A = \hat{A}A^{(\text{Tot,WT})}.$$

bioRxiv preprint doi: https://doi.org/10.1101/2023.08.04.552031; this version posted August 28, 2024. The copyright holder for this preprint (which was not certified by peer review) is the author/funder, who has granted bioRxiv a license to display the preprint in perpetuity. It is made available under aCC-BY 4.0 International license.

Substituting into (M1) gives dynamics in non-dimensional form [6, Eq. 14]

$$\partial_{\hat{t}} \hat{A}_1 = \hat{D}_1 \partial_{\hat{x}}^2 \hat{A}_1 + \hat{K}_{\text{on}} \left(1 + \hat{K}_{\text{f}} \hat{F}(\hat{A})\right) \hat{A}_{\text{c}} - \hat{K}_{\text{off}} \hat{A}_1$$

$$+ 2\hat{A}_2 - 2\hat{K}_{\text{p}} \hat{A}_1{}^2 + \sum_{n=3}^{N} \left(\hat{A}_n - \hat{K}_{\text{p}} \hat{A}_1 \hat{A}_{n-1}\right) \tag{M2a}$$

$$\partial_{\hat{t}} \hat{A}_n = \hat{K}_{\text{p}} \hat{A}_1 (\hat{A}_{n-1} - \hat{A}_n) - (\hat{A}_n - \hat{A}_{n+1}) \qquad N > n \geq 2 \tag{M2b}$$

$$\partial_t \hat{A}_N = \hat{K}_{\text{p}} \hat{A}_1 \hat{A}_{N-1} - \hat{A}_N \tag{M2c}$$

$$\hat{A}_{\text{c}} = \hat{A}^{(\text{Tot})} - \int_0^1 \hat{A}(x)\, d\hat{x}, \tag{M2d}$$

Equations (M2) involve six dimensionless parameters

$$\hat{D}_1 = \frac{D_1}{L^2 k_{\text{dep}}}, \quad \hat{K}_{\text{on}} = \frac{k_{\text{basal}}}{k_{\text{dep}} h}, \quad \hat{K}_{\text{f}} = \frac{k_f A^{(\text{Tot,WT})}}{k_{\text{basal}}}, \tag{M2e}$$

$$\hat{K}_{\text{off}} = \frac{k_{\text{off}}}{k_{\text{dep}}}, \quad \hat{K}_{\text{p}} = \frac{k_{\text{pol}} A^{(\text{Tot,WT})}}{k_{\text{dep}}}, \quad \hat{A}^{(\text{Tot})} = \frac{A^{(\text{Tot})}}{A^{(\text{Tot,WT})}}, \quad \hat{F}(\hat{A}) = \frac{f(A)}{A^{(\text{Tot,WT})}}.$$

(and the feedback function $F(\hat{A})$; see below), whose values we determine by direct measurement or by fitting to experimental data. Our data directly give values for three dimensional parameters: the monomeric diffusion coefficient $D_1 = 0.1$ μm^2/s, the monomer detachment rate $k_{\text{off}} = 3$/s, and the rate at which single subunits unbind from PAR-3 oligomers (0.08/s), which gives $k_{\text{dep}} = 0.16$/s (since unbinding can occur from either side). We take the length scale $L = 134.6$ μm to be the perimeter length of the maximal cross-section of an ellipsoid, with half-axis lengths $27 \times 15 \times 15$ μm, which approximates the shape and dimensions of a typical *C. elegans* embryo in mitosis [2]. Together, these measurements determine values for the non-dimensional parameters $\hat{D}_1$ and $\hat{K}_{\text{off}}$. The parameter $\hat{A}^{(\text{Tot})}$ represents a depletion factor; it is 1 in wild-type embryos and later varied to mimic depletion of PAR-3.

1.1 Oligomerization and effective dissociation

To fit the polymerization rate $\hat{K}_{\text{p}}$, we first solve (M2) at steady state to obtain an exponential distribution of oligomer sizes [1, 6]

$$\hat{A}_n = \hat{K}_{\text{p}} \hat{A}_1 \hat{A}_{n-1} := \alpha \hat{A}_{n-1} \quad n \geq 2. \tag{M3}$$

This defines $\alpha = \hat{K}_{\text{p}} \hat{A}_1 = (k_{\text{pol}}/k_{\text{dep}})\, A_1$ as the coefficient of the exponential distribution of oligomer sizes. It follows that the total amount of PAR-3 is given by

$$\hat{A} = \sum_{n=1}^{N} n\alpha^{n-1} \hat{A}_1 \xrightarrow{N \to \infty} \frac{\hat{A}_1}{(1-\alpha)^2}. \tag{M4}$$

bioRxiv preprint doi: https://doi.org/10.1101/2023.08.04.552031; this version posted August 28, 2024. The copyright holder for this preprint (which was not certified by peer review) is the author/funder, who has granted bioRxiv a license to display the preprint in perpetuity. It is made available under aCC-BY 4.0 International license.

This equation can then be solved for $\hat{A}_1$ to obtain [6, Eq. (12)]

$$\hat{A}_1 = \frac{1 + 2\hat{A}\hat{K}_{\mathrm{p}} - \sqrt{1 + 4\hat{K}_{\mathrm{p}}\hat{A}}}{2\hat{A}\left(\hat{K}_{\mathrm{p}}\right)^2}, \tag{M5}$$

which gives the number of bound monomers (and therefore α) as a function[1] of the total bound $\hat{A}$. To fit the value of $\hat{K}_{\mathrm{p}}$, we use our measurements from the polarized state in *par-1* mutant embryos (Fig. 3). Our particular assumption is that there is roughly 25% bound PAR-3 during maintenance phase,[2] which gives $\hat{A} \approx 0.4$ on the anterior. Combining this with our measurement $\alpha = 0.73$ on the anterior cortex (Fig. 3H), we choose $\hat{K}_{\mathrm{p}}$ such that $\alpha\left(\hat{K}_{\mathrm{p}}, \hat{A} = 0.4\right) = 0.73$, which gives an estimate $\hat{K}_{\mathrm{p}} = 25$. The corresponding value of α on the posterior (where $\hat{A} = 0.4/8 = 0.05$) is predicted to be $\alpha\left(\hat{K}_{\mathrm{p}} = 25, \hat{A} = 0.05\right) = 0.42$, in precise agreement with experimental observations.

Once $\hat{K}_{\mathrm{p}}$ is known, we can compute the effective dissociation rate (Fig. 4E) by assuming that the distribution of oligomer sizes is given by (M3), with detachment rates given by $k_{\mathrm{off}}^{(n)} = k_{\mathrm{off}}^{(1)}\beta^{n-1}$. This gives the effective dissociation rate

$$k_{\mathrm{effective}} = \frac{k_{\mathrm{off}}^{(1)}\hat{A}_1}{\hat{A}}\sum_{n=1}^{\infty} n\alpha^{n-1}\beta^{n-1} = \frac{k_{\mathrm{off}}^{(1)}\hat{A}_1}{\hat{A}\left(1-\alpha\beta\right)^2} = k_{\mathrm{off}}^{(1)}\left(\frac{1-\alpha}{1-\alpha\beta}\right)^2, \tag{M6}$$

where $\alpha(\hat{A}) = \hat{A}_1(\hat{A})\hat{K}_{\mathrm{p}}$ is obtained using (M3) again. This results is shown in Fig. 4E; note however that using an exponential relationship for the oligomer sizes and a nonzero detachment rate for oligomer sizes larger than one is inconsistent at steady state.

1.2 The form of the feedback strength and resulting steady states

To fit the remaining dimensionless parameters[3] $\hat{K}_{\mathrm{on}}$ and $\hat{K}_{\mathrm{f}}$, we seek values for which model predictions match steady state behaviors observed in wild type and *spd-5(RNAi)* embryos. First, because PAR-3 distributions remain roughly uniform throughout polarity establishment and maintenance

[1]This convenient closed-form expression is a consequence of our neglect of dimer and trimer unbinding. If dimer and trimer unbinding are included, then (M3) only holds for $n \geq 4$, and $\hat{A}_1$ can be obtained from $\hat{A}$ by solving a quartic equation. The resulting model has negligible quantitative difference (at most 20% in the predicted recruitment asymmetry in polarized states) from the one we present here, which is significantly easier to analyze.

[2]This number is similar to that measured for PAR-2 and PAR-6 in [3, Fig. S3]. Considering our measurements (Fig. 2) reveal that roughly half of the bound PAR-3 leaves the membrane after establishment, the maximum percentage of bound protein is 50%, and we take half this number as an estimate. In any case, the results of our model do not change when we change this (arbitrary) assumption.

[3]The parameter h (cytoplasmic "thickness") only appears in the dimensionless parameter $\hat{K}_{\mathrm{on}}$. Since we fit $\hat{K}_{\mathrm{on}}$, we never assign a value to h in Table M1.

bioRxiv preprint doi: https://doi.org/10.1101/2023.08.04.552031; this version posted August 28, 2024. The copyright holder for this preprint (which was not certified by peer review) is the author/funder, who has granted bioRxiv a license to display the preprint in perpetuity. It is made available under aCC-BY 4.0 International license.

phases in embryos lacking a functional sperm cue or myosin function, we infer that the spatially uniform steady state is stable. Second, because the polarized state remains stable through maintenance phase in *par-1* mutant embryos, we infer that in the absence of posterior inhibition by PAR-1, PAR-3 dynamics encode the coexistence of spatially uniform and spatially non-uniform (polarized) states. Third, both experiments and simulations show that in the polarized state, the boundary position is stable on timescales relevant to maintenance phase, i.e., the effects of diffusion can be ignored in comparing observed and predicted polarized states. Finally, we observe that the density of membrane bound PAR-3 observed on the anterior cortex during maintenance phase in wild type or *par-1* mutant embryos is roughly the same as that observed throughout the cortex in *spd-5(RNAi)* embryos (Fig. S1) and about eight times that observed on the posterior pole of *par-1* mutant embryos. Thus we seek parameter values satisfying two conditions:

C1: A stable spatially uniform steady state with density $\hat{A}_u$ coexists with a stable spatially non-uniform steady state, characterized by $\hat{A} = \hat{A}_h$ on 50% domain length and $\hat{A} = \hat{A}_\ell$ on the other 50%.

C2: $\hat{A}_h/\hat{A}_\ell \approx 8$ and $\hat{A}_h/\hat{A}_u \approx 1$.

Because model predictions depend upon the form of positive feedback, (encoded by the function $F(\hat{A})$), we next describe the fitting approach and qualitative results for different assumptions about feedback. In all cases, we focus on predicted behavior at/near the spatially uniform and polarized steady states. Neglecting diffusion, and invoking the steady state (M3) for polymerization dynamics reduces the monomer equation (M2a) to the balance of an attachment flux $\delta(\hat{A})$ (which sums the fluxes due to basal attachment and feedback) and a detachment flux $\nu(\hat{A})$,

$$0 = \underbrace{\hat{K}_{\text{on}} \left(1 + \hat{K}_{\text{f}} \hat{F}^{+}(\hat{A})\right) \hat{A}_{\text{c}}}_{\text{Attachment, } \delta(\hat{A})} - \underbrace{\hat{K}_{\text{off}} \hat{A}_1(\hat{A})}_{\text{Detachment, } \nu(\hat{A})} \tag{M7}$$

where $\hat{A}_1(\hat{A})$ is given in (M5). At spatially uniform steady states, $\hat{A}(x) \equiv \hat{A}$, the dimensionless cytoplasmic concentration (defined in (M2d)) becomes $\hat{A}_{\text{c}} = \hat{A}^{(\text{Tot})} - \hat{A}$ (recall $\hat{A}^{(\text{Tot})} = 1$ for wild type embryos), and the total attachment rate is

$$\delta(\hat{A}) = \delta_{\text{cons}}(\hat{A}) = \hat{K}_{\text{on}} \left(1 + \hat{K}_{\text{f}} \hat{F}^{+}(\hat{A})\right) \left(1 - \hat{A}\right) \tag{M8}$$

In this case, the attachment and detachment curves in (M7) depend only on $\hat{A}$, and spatially uniform steady state(s) can be read from their intersections on a flux balance plot (solid red and

bioRxiv preprint doi: https://doi.org/10.1101/2023.08.04.552031; this version posted August 28, 2024. The copyright holder for this preprint (which was not certified by peer review) is the author/funder, who has granted bioRxiv a license to display the preprint in perpetuity. It is made available under aCC-BY 4.0 International license.

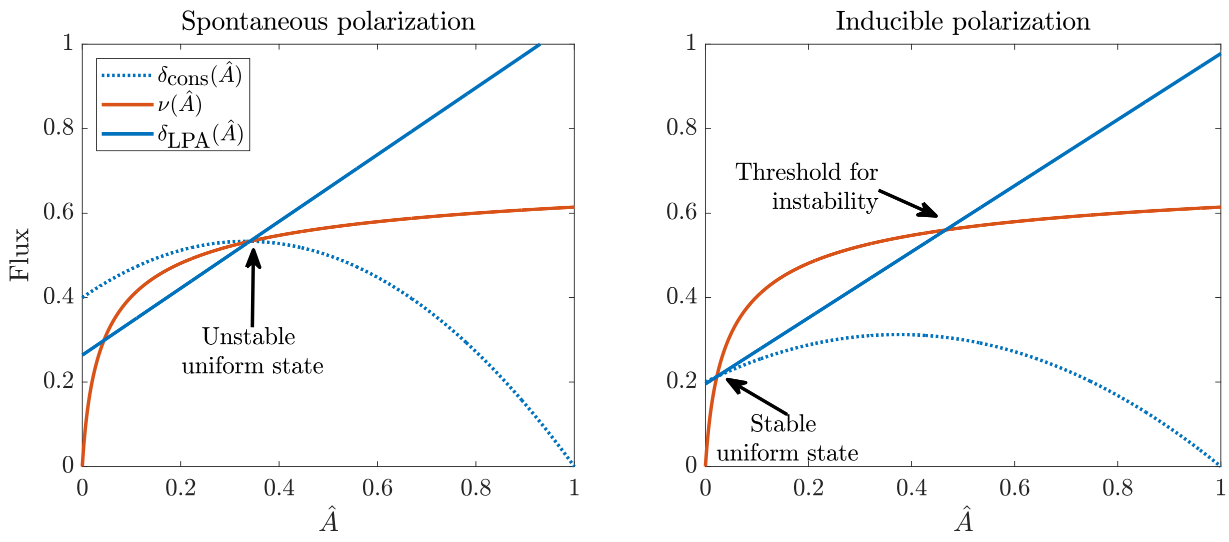

Figure M1: Flux plane analysis for linear feedback model (M10). The uniform state is defined by the single crossing of the attachment rate $\delta_{\text{cons}}(\hat{A})$ (dotted blue lines, see (M8)) with the detachment rate $\nu(\hat{A})$ (solid red lines). *Stability* of the uniform state is determined by how the attachment rate $\delta_{\text{LPA}}(\hat{A})$ (solid blue lines, see (M9)) compares to $\nu(\hat{A})$ near the steady state. The left panel demonstrates an unstable uniform state, while the right panel demonstrates a stable uniform state with inducible polarization, defined as having the threshold for instability at $\hat{A} \leq 2$.

dotted blue lines in Fig. M1). Following the Local Perturbation Analysis (LPA) approach of [4], we determine stability by considering the response to a local perturbation $\hat{A} = \hat{A}_u + \epsilon\hat{A}$ of finite size within an infinitesimally small domain such that the cytoplasmic concentration remains constant ($\hat{A}_c = 1 - \hat{A}_u$). This situation corresponds to an attachment rate of

$$\delta(\hat{A}) = \delta_{\text{LPA}}(\hat{A}) = \hat{K}_{\text{on}}\left(1 + \hat{K}_{\text{f}}\hat{F}^{+}(\hat{A})\right)\left(1 - \hat{A}_u\right). \tag{M9}$$

The qualitative response of the system can be inferred by examining the behavior of $\Delta(\hat{A}) = \delta_{\text{LPA}}(\hat{A}) - \nu(\hat{A})$. The case when $\Delta'(\hat{A}_u) > 0$ corresponds to an unstable uniform state, while $\Delta'(\hat{A}_u) < 0$ corresponds to a stable one.

1.3 Linear feedback model

We first consider the linear feedback model that was used previously by some of us [6]

$$f(A) = A \to \hat{F}(\hat{A}) = \hat{A}. \tag{M10}$$

Using LPA, we determine the qualitative behavior for different values of the two unknown parameters: the basal attachment rate $\hat{K}_{\text{on}}$ and the feedback strength $\hat{K}_{\text{f}}$. Varying feedback strength

bioRxiv preprint doi: https://doi.org/10.1101/2023.08.04.552031; this version posted August 28, 2024. The copyright holder for this preprint (which was not certified by peer review) is the author/funder, who has granted bioRxiv a license to display the preprint in perpetuity. It is made available under aCC-BY 4.0 International license.

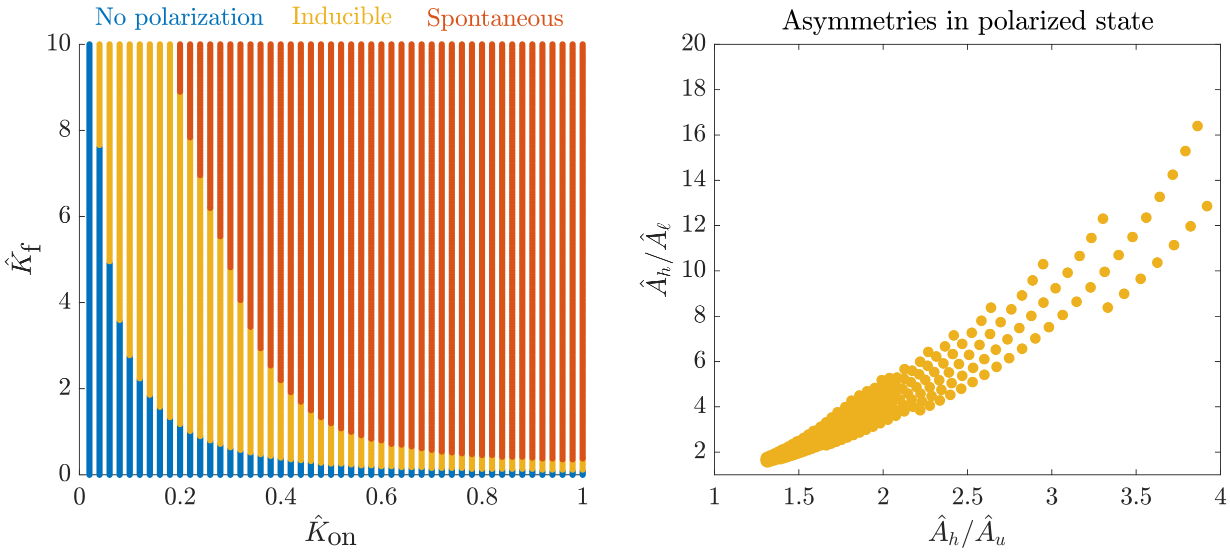

Figure M2: Parameterizing the linear feedback model. At left, we show the phase diagram for the two unknown parameters, $\hat{K}_{\mathrm{on}}$ and $\hat{K}_{\mathrm{f}}$, and break the dynamics into the three regimes of no polarization, inducible polarization, and spontaneous symmetry breaking. Then, at right, we simulate a polarized state with the parameters in the inducible regime. We simulate starting with a 10:1 protein asymmetry with 50% bound protein, then plot the ratios $\hat{A}_h/\hat{A}_u$ and $\hat{A}_h/A_\ell$ after five minutes.

for any fixed basal attachment rate, the qualitative behavior of this system falls into one of three classes (see Figs. M1 and M2):

1. For sufficiently large $\hat{K}_{\mathrm{f}}$, the spatially uniform state is unstable to fluctuations or perturbations of any size (left panel of Fig. M1).

2. For intermediate $\hat{K}_{\mathrm{f}}$, the spatially uniform state is stable to small perturbations, but it is possible to induce polarization by driving the local concentration above a threshold, which is given by a second crossing of attachment and detachment curves in the flux balance plot.

3. Finally, as $\hat{K}_{\mathrm{f}}$ approaches 0, the threshold to induce polarization approaches ∞, and it is essentially impossible to induce polarization.

For the linear model, the phase diagram for polarization (Figure M2) shows that polarity is inducible (a quasi-stable spatially non-uniform steady state exists) for a subset of model parameters. For each reasonable choice of these parameters (specifically, choices which yield a uniform state with mean oligomer size between 2.5 and 3.5), we determine the polarized "steady state" from asymmetric initial conditions (10:1 initial asymmetry on 50% of the domain with 50% of the total protein initially bound) by simulating for five minutes and evaluating the ratios $\hat{A}_h/\hat{A}_\ell$ and $\hat{A}_h/\hat{A}_u$.

bioRxiv preprint doi: https://doi.org/10.1101/2023.08.04.552031; this version posted August 28, 2024. The copyright holder for this preprint (which was not certified by peer review) is the author/funder, who has granted bioRxiv a license to display the preprint in perpetuity. It is made available under aCC-BY 4.0 International license.

We find that none of the parameter values yielding inducible polarity give ratios of $\hat{A}_h/\hat{A}_\ell$ and $\hat{A}_h/\hat{A}_u$ that match those observed empirically. In essence, for the linear model to achieve five-to-ten-fold ratio of densities in the non-uniform steady state, the higher density must be significantly (2 to 3.5 times) higher than that observed in the spatially uniform steady state, since it must be large enough to overcome the threshold required to induce polarization (this is the scenario depicted in the right panel of Fig. M1).

1.4 Saturated feedback model

Our experimental finding that PAR-3 recruitment asymmetries do not change during maintenance phase as levels of membrane-bound PAR-3 fall by $\approx 50\%$ suggest that positive feedback saturates at or below the density observed in late maintenance. Thus, we consider a simple model for saturating feedback given by

$$f(A) = \min\left(A, A^{(\text{Sat})}\right) \to \hat{F}(\hat{A}) = \min\left(\hat{A}, \hat{A}^{(\text{Sat})}\right), \tag{M11}$$

where $\hat{A}^{(\text{Sat})} < \hat{A}_u$.

As in the case for linear feedback, we use the saturated feedback function (M11) to identify spatially uniform steady states as solutions of the flux balance equation (M7) with $\delta = \delta_{\text{cons}}(\hat{A})$ given in (M8). We then evaluate the stability of the uniform state using LPA; as shown in the flux balance plot in Fig. M3, if the feedback saturates below the spatially uniform steady state concentration, the spatially uniform steady state is always stable. However, for a subset of values of ($\hat{K}_{\text{on}}$, $\hat{K}_{\text{f}}$ and $\hat{A}^{(\text{Sat})}$), polarity can be induced by local perturbations that drive $\hat{A}$ *below* some threshold. In this case, at the stably polarized steady state, $\hat{A}_\ell$ lies below the threshold for inducing polarity, while $\hat{A}_h$ is held close to spatially uniform density $\hat{A}_u$ by saturating feedback, making it possible to satisfy conditions C1 and C2 ($\hat{A}_h/\hat{A}_\ell \approx 8$ and $\hat{A}_h/\hat{A}_u \approx 1$) from above.

To select values for $\hat{K}_{\text{on}}$, $\hat{K}_{\text{f}}$ and $\hat{A}^{(\text{Sat})}$, we first observe that $\hat{A}^{(\text{Sat})}$ cannot lie too far below $\hat{A}_h$, since otherwise the threshold crossing that defines inducible polarity in the LPA flux balance curves (Fig. M3) cannot occur. We therefore begin by setting the saturation threshold at $\hat{A}^{(\text{Sat})} = 0.8\hat{A}_u$. Then, we scan over all pairs of $\left(\hat{K}_{\text{on}}, \hat{K}_{\text{f}}\right)$ to find a polarized state with anterior concentration $\hat{A}_h \approx 0.4$ (which, because of our fitting of the depolymerization curve, gives $\alpha = 0.73$ on the anterior) and an A/P ratio of approximately 8. As in the linear feedback model, the scan happens in two steps (Fig. M4): we first make a phase diagram separating into regimes with no polarization and bistable dynamics. Then, we study the polarized states by repeating the same set of simulations we used for the linear model, where we evolve an initial 10:1 asymmetry in time and plot the ratios

bioRxiv preprint doi: https://doi.org/10.1101/2023.08.04.552031; this version posted August 28, 2024. The copyright holder for this preprint (which was not certified by peer review) is the author/funder, who has granted bioRxiv a license to display the preprint in perpetuity. It is made available under aCC-BY 4.0 International license.

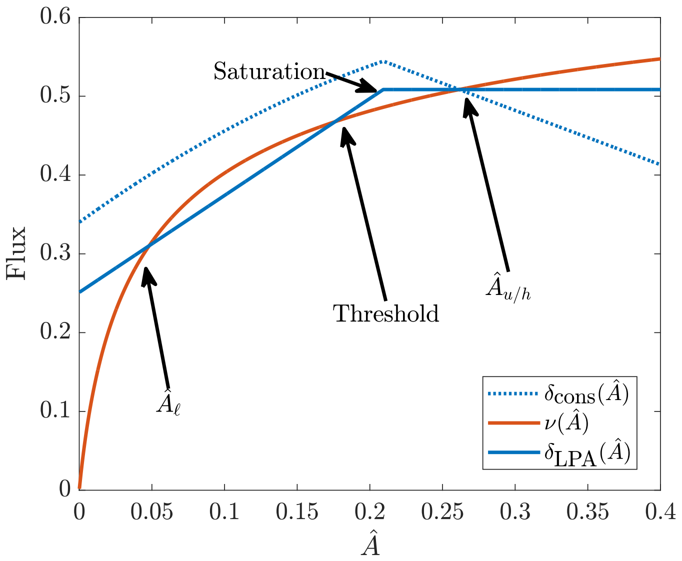

Figure M3: Flux plane analysis for linear feedback model with saturation at 80% of uniform state. As in Fig. M1, the uniform state is determined by the crossing of $\delta_{\mathrm{cons}}(\hat{A})$ and $\nu(\hat{A})$, while the stability of the uniform state is determined by $\delta_{\mathrm{LPA}}(\hat{A})$. The uniform state is stable if the feedback saturates below it.

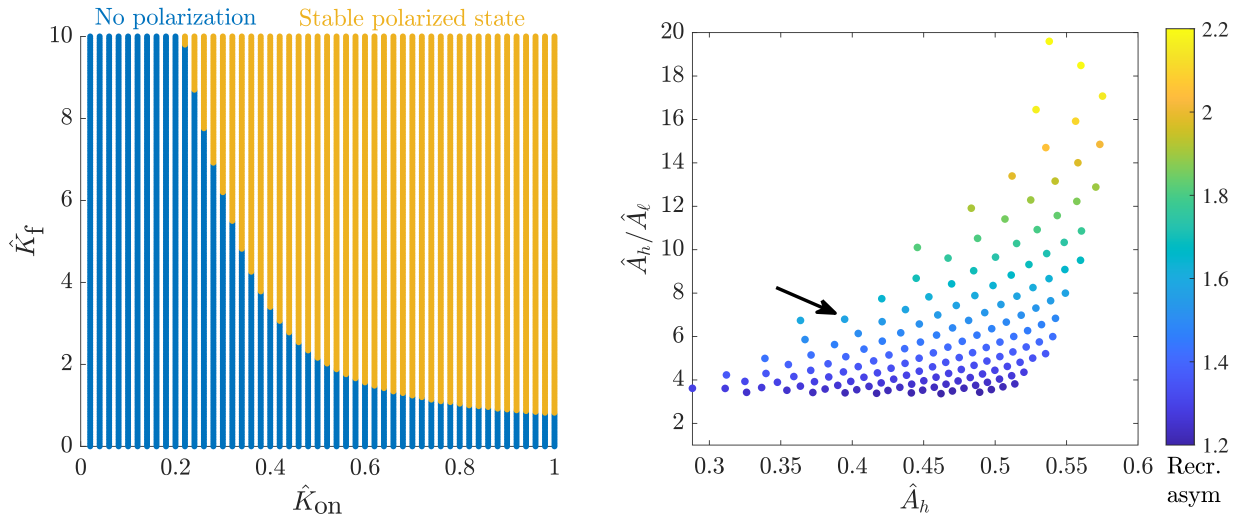

Figure M4: Parameterizing the linear feedback model with saturation, given $\hat{A}^{(\mathrm{Sat})} = 0.8\hat{A}_u$. (Left:) Phase diagram for the two unknown parameters, $\hat{K}_{\mathrm{on}}$ and $\hat{K}_{\mathrm{f}}$. We break the dynamics into the two regimes of no polarization (one crossing of $\delta_{\mathrm{LPA}}(\hat{A})$ and $\nu(\hat{A})$ in the flux balance plot), and bistable dynamics (three crossings). (Right:) We simulate the polarized state by starting from a 10:1 initial asymmetry on 50% of the domain with 50% of the total protein bound, then running forward in time. We plot the ratios $\hat{A}_h/\hat{A}_u$ and $\hat{A}_h/A_\ell$, colored by the recruitment asymmetry, $\delta(\hat{A}_h)/\delta(A_\ell)$, all after five minutes. Black arrow shows our chosen parameters.

bioRxiv preprint doi: https://doi.org/10.1101/2023.08.04.552031; this version posted August 28, 2024. The copyright holder for this preprint (which was not certified by peer review) is the author/funder, who has granted bioRxiv a license to display the preprint in perpetuity. It is made available under aCC-BY 4.0 International license.

Parameter	Description	Value	Units	Ref	Notes
L	Domain length	134.6	μm	[2]	radii 27×15 μm ellipse
D_1	Monomeric PAR-3 diffusivity	0.1	μm^2/s		This study
$\hat{K}_{\text{on}}$	Monomeric PAR-3 attachment rate	0.34			Fitting
$\hat{K}_{\text{f}}$	Feedback strength	4.9			Fitting
$\hat{A}^{(\text{Sat})}$	Feedback saturation level	0.21			80% of uniform state in wild type
k_{off}	Monomeric PAR-3 detachment rate	3	1/s		This study, Fig. 3K
k_{dep}	PAR-3 depolymerization rate	0.16	1/s		This study, Fig. 4E
$\hat{K}_{\text{p}}$	PAR-3 polymerization rate	25			Fit $\alpha = 0.73$ with $\hat{A} = 0.4$
N	Max oligomer size	50			Same results for larger N

Table M1: Parameter values for the PAR-3 model (see (M2e) for the definitions of dimensionless parameters).

$\hat{A}_h/\hat{A}_u$ and $\hat{A}_h/A_\ell$ after five minutes (again only simulating with parameters that have a mean oligomer size between 2.5 and 3.5 in the uniform state). Fig. M4 shows that a choice of parameters ($\hat{K}_{\text{on}} = 0.34$, $\hat{K}_{\text{f}} = 4.9$) exists which satisfies $\hat{A}_h = 0.4$ with an A/P ratio of 7. This choice gives a recruitment asymmetry of 1.6 after 5 minutes, which is about 80% of the value we measured experimentally, and a wild-type uniform state of $\hat{A}_u = 0.26$, with corresponding feedback saturation level $\hat{A}^{(\text{Sat})} = 0.8\hat{A}_u \approx 0.21$. Table M1 summarizes the parameters.

We note that the (arbitrary) choice of saturation level and anterior concentration $\hat{A}_h$ make little difference in our model, as changes in these parameters simply give different choices of $\hat{K}_{\text{on}}$ and $\hat{K}_{\text{f}}$ that satisfy the desired conditions. More fundamentally, the saturating feedback model gives a ratio $\hat{A}_h/\hat{A}_u \approx 1.5$, so that the anterior state concentration exceeds that of the uniform state by only 50%, relative to 100–200% in the linear feedback model. Unlike in the linear model, where such a change was necessary to surpass the threshold for polarization, here the change is just a consequence of a higher cytoplasmic concentration (and thus a higher attachment rate, which comes into equilibrium with the nonlinear detachment curve).

1.5 Phase diagram

In order to compare the model to experimental data, we use simulations of the polarized state to construct a phase diagram of the stability behavior as a function of the mean oligomer size and feedback strength in Fig. 7B. To generate the phase diagram, we vary $\hat{K}_{\text{p}}$, $\hat{K}_{\text{on}}$, and $\hat{K}_{\text{f}}$, compute the uniform state, and assign the feedback saturation level. Simulated states are defined to be

bioRxiv preprint doi: https://doi.org/10.1101/2023.08.04.552031; this version posted August 28, 2024. The copyright holder for this preprint (which was not certified by peer review) is the author/funder, who has granted bioRxiv a license to display the preprint in perpetuity. It is made available under aCC-BY 4.0 International license.

"polarized" if the A/P ratio is larger than 2 after $t = 5$ mins of simulation time. Data points are plotted based on the "feedback strength"

$$\text{Feedback strength} = \hat{K}_\text{f}\hat{F}^+(\hat{A}) = \frac{k_f}{k_\text{basal}} \min\left(\hat{A}, \hat{A}^{(\text{Sat})}\right) = \frac{k_f \hat{A}^{(\text{Sat})}}{k_\text{basal}},$$

(the last equality holds since in the polarized state $\hat{A}_h > \hat{A}_u > \hat{A}^{(\text{Sat})}$), and mean oligomer size

$$\text{Mean oligomer size} = \left(\sum_{n=1}^{N} n\hat{A}_n\right) / \left(\sum_{n=1}^{N} \hat{A}_n\right),$$

at $t = 5$ mins. The phase diagram shows that bistability is possible at smaller feedback strengths if the mean oligomer size is large enough, and vice versa.

2 Dynamics

We use standard numerical methods to solve (M2), discretizing the one-dimensional domain at N points, with diffusion being discretized via the standard three point Laplacian. We use a forward Euler method for the reaction terms, and a backward Euler method for the diffusion terms (in the monomer equation), which gives first order accuracy in time [5, c. 1]. Typical parameters are $N = 1000$ (required to resolve the boundary layer where the enriched PAR-3 domain can shift) and $\Delta\hat{t} = 0.02$ (50 steps per depolymerization time). Matlab code is available at the github repository `https://github.com/omaxian/CElegansModel/`.

We first simulate our PAR-3 model with two different initial conditions (Fig. M5): a perturbation of the uniform state (which demonstrates that the uniform state is a stable attractor) and a polarized state with all protein initially bound, which decays to a stable polarized state. In both cases, the timescale on which the protein levels adjust is roughly 5–10 minutes.

2.1 Slow drift of the boundary

In our simulation of the polarized state in Fig. M5, we notice the appearance of a drift of the boundary in time. This drift, which is controlled by diffusion, appears to be much slower than the equilibration of concentration. To understand it in more detail, in Fig. M6 we plot the evolution of a polarized PAR-3 domain of various initial sizes (beginning with a 10:1 asymmetry and 50% protein bound), recording the ratio $\hat{A}_h/\hat{A}_\ell$, as well as the overall domain size. There we see rapid (timescale roughly 5–10 minutes) equilibration of the initial concentrations, followed by a slow drift (timescale much longer than 60 minutes) of the boundary to a unique position. Note that even

bioRxiv preprint doi: https://doi.org/10.1101/2023.08.04.552031; this version posted August 28, 2024. The copyright holder for this preprint (which was not certified by peer review) is the author/funder, who has granted bioRxiv a license to display the preprint in perpetuity. It is made available under aCC-BY 4.0 International license.

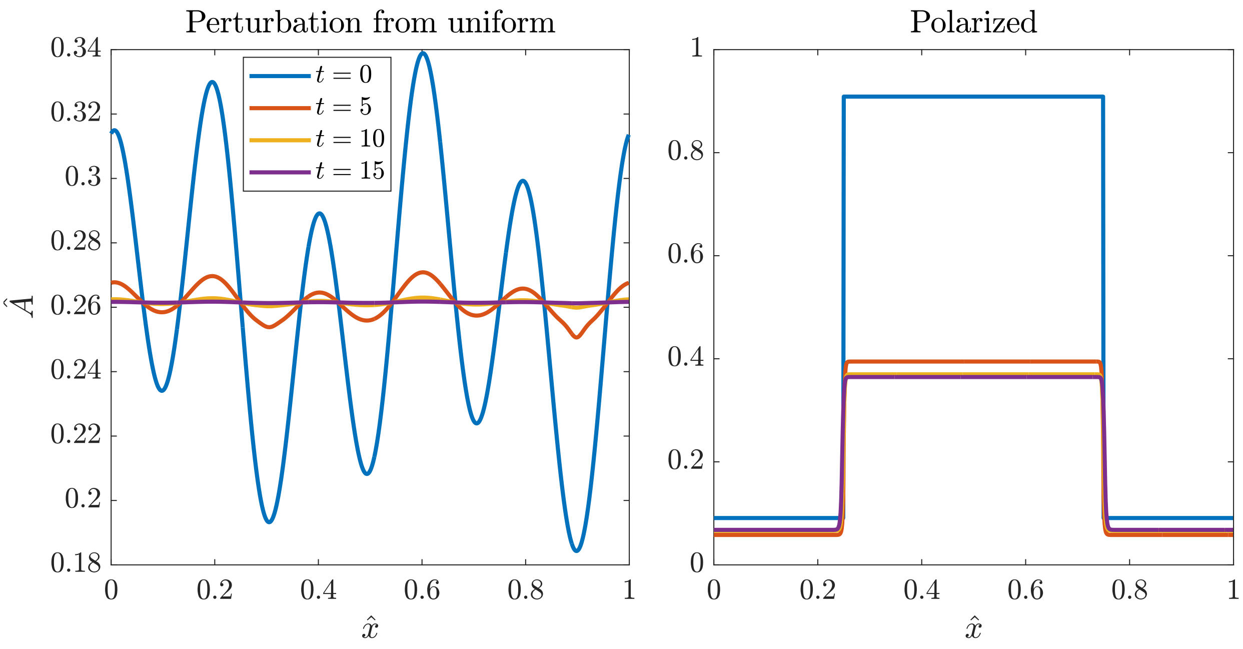

Figure M5: Dynamics of the saturated feedback model. At left, we show that perturbations to the uniform state decay, while at right, we show that the polarized state is stable in time. Dynamics occur on timescales of 5–10 minutes (legend).

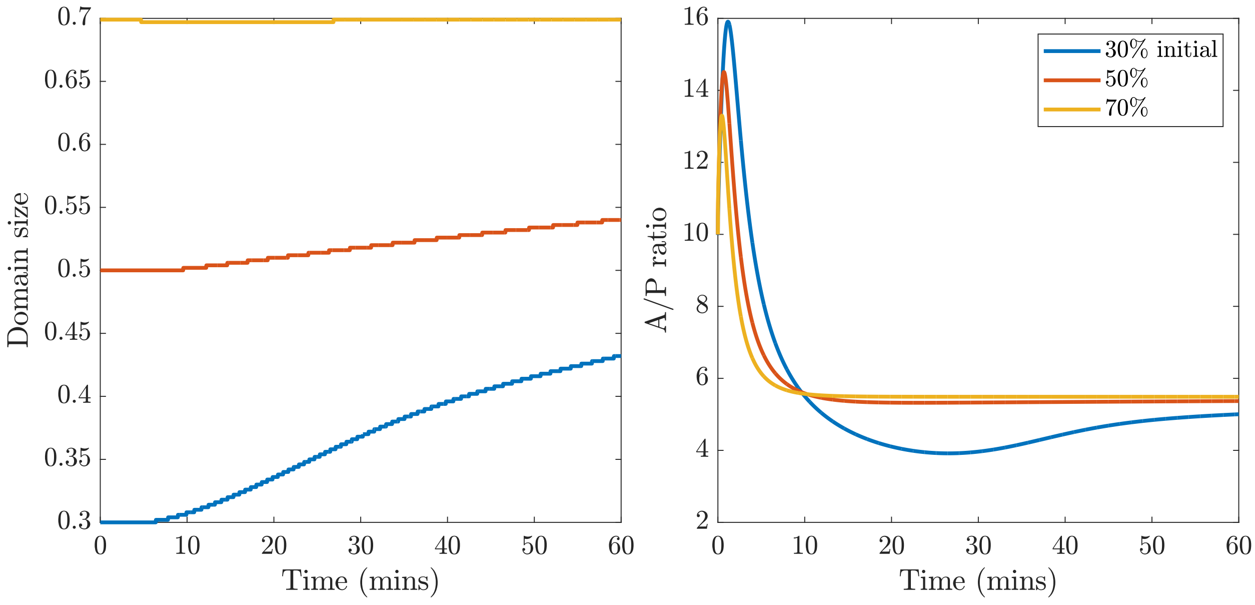

Figure M6: Dynamics of an initially polarized domain. We consider initial enriched domain sizes of 30, 50, and 70%, and impose a 10:1 asymmetry at $t = 0$ with 50% protein bound to the membrane to mimic the end of establishment phase. We then track the enriched domain size and $\hat{A}_h/\hat{A}_\ell$ over time. There is a relatively fast timescale of concentration equilibration, followed by slow boundary drift.

bioRxiv preprint doi: https://doi.org/10.1101/2023.08.04.552031; this version posted August 28, 2024. The copyright holder for this preprint (which was not certified by peer review) is the author/funder, who has granted bioRxiv a license to display the preprint in perpetuity. It is made available under aCC-BY 4.0 International license.

the "fast" shift in concentrations, which occurs on a scale of 5–10 minutes, is still slow enough to compete with the timescale of maintenance phase. That is, even at the end of maintenance phase, the concentrations are still likely transitioning to the steady state.

2.2 Systematic depletion of PAR-3

To model the experiment of PAR-3 depletion (Fig. 7), we vary the total fraction of PAR-3 $\hat{A}^{(\mathrm{Tot})}$. To simulate experimental conditions for a given $\hat{A}^{(\mathrm{Tot})}$, we start at the "end of establishment phase," which corresponds to 50% protein bound to the membrane and a roughly 10:1 ratio of anterior to posterior protein and an anterior domain size of 50% (this the dark blue protein profile in the right panel of Figs. M5). We then run forward in time until we reach $t = 4$ minutes, corresponding exactly to the time interval used in experiments, after which we record the mean oligomer size on the enriched domain, and the A/P ratio. Because the dimensionless parameters in (M2e) are in terms of the total PAR-3 in *wild-type* embryos, only $\hat{A}^{(\mathrm{Tot})}$ changes when we perform the depletion experiment.

The results in Fig. 7 show that our model successfully reproduces the trend in mean oligomer size vs. total bound density (on the anterior). For the A/P asymmetries, our model captures the qualitative and quantitative trend of no asymmetry for low mean oligomer sizes, followed by a transition to stable asymmetries on the order 6–8 for larger mean oligomer sizes. This is in contrast to a model based on asymmetric recruitment (in which case there is no feedback and monomers are recruited to the anterior at 1.7 times the rate of the posterior), which predicts a persistent asymmetry even as the mean oligomer size drops to 1 (i.e., even without oligomerization).

2.3 Changing feedback to mimic aPAR depletion

Finally, we use our saturated feedback model to demonstrate what might happen when the feedback is reduced, in order to explain the results of some of our perturbation experiments. To do this, we perturb the feedback parameter $\hat{K}_\mathrm{f}$ and examine changes to the flux balance plot (Fig. M7) and dynamics of an initially polarized state (10:1 asymmetry with 50% protein bound; main text Fig. 8). We are already familiar with the flux balance plot for wild type parameters (Fig. M3). Simulating maintenance phase with these parameters yields the expected dynamics: the anterior domain concentration decreases, while the posterior remains roughly constant, and there is a steady state asymmetry (solid lines in the bottom panel).

Reducing to 0% feedback ($\hat{K}_\mathrm{f} = 0$) gives dynamics which correspond roughly to our experiment

bioRxiv preprint doi: https://doi.org/10.1101/2023.08.04.552031; this version posted August 28, 2024. The copyright holder for this preprint (which was not certified by peer review) is the author/funder, who has granted bioRxiv a license to display the preprint in perpetuity. It is made available under aCC-BY 4.0 International license.

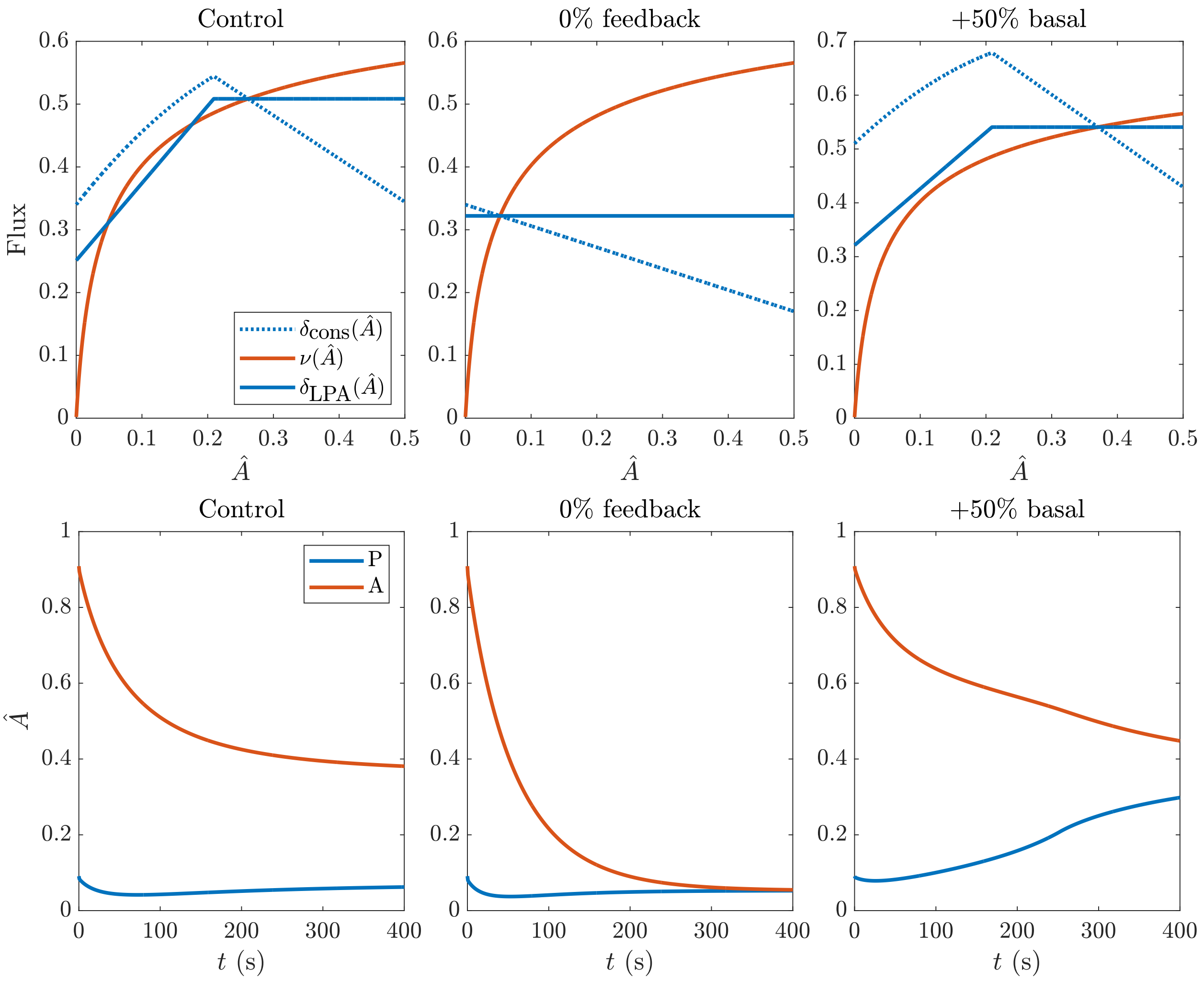

Figure M7: How the system responds to reductions in feedback and changes in the basal rate. Top plots: theoretical flux balance when considering default parameters ($\hat{K}_{\text{f}} = 4.9$, $\hat{K}_{\text{on}} = 0.34$), 0% feedback ($\hat{K}_{\text{f}} = 0$, $\hat{K}_{\text{on}} = 0.34$), and 150% basal binding rate ($\hat{K}_{\text{f}} = 3.3$, $\hat{K}_{\text{on}} = 0.51$). Bottom plot: trajectory of an initially 10:1 polarized state (split over half the embryo length with 50% bound protein) over time.

bioRxiv preprint doi: https://doi.org/10.1101/2023.08.04.552031; this version posted August 28, 2024. The copyright holder for this preprint (which was not certified by peer review) is the author/funder, who has granted bioRxiv a license to display the preprint in perpetuity. It is made available under aCC-BY 4.0 International license.

with PAR-6 depletion (RNAi). In this case, removing feedback gives a single, smaller, uniform steady state which is close to posterior levels in the wild type. In simulations (Fig. 8), we consequently observe a convergence of the anterior and posterior levels onto a smaller concentration, near to the original posterior level.

For CDC-42 depletion (RNAi), we hypothesize that an increase in the basal binding rate could lead to a loss of asymmetry. To test this, we increase k_{basal} by 50%; using the non-dimensionalization in (M2), this implies $\hat{K}_{\text{on}}$ increases by 50% and $\hat{K}_{\text{f}}$ decreases by 33%. As shown in the top right panel in Fig. M7, the effect of this change is to increase the y intercept of the local flux balance curve δ_{LPA}, which leads to a loss of bistability. As shown in the bottom right panel of Fig. M7, the dynamics with this choice of parameters roughly correspond to our experiment in CDC-42 (RNAi) embryos, where the posterior state rises up to meet the anterior one.

References

[1] Leah Edelstein-Keshet and G Bard Ermentrout. Models for the length distributions of actin filaments: I. simple polymerization and fragmentation. *Bulletin of mathematical biology*, 60(3):449–475, 1998.

[2] Nathan W Goehring, Philipp Khuc Trong, Justin S Bois, Debanjan Chowdhury, Ernesto M Nicola, Anthony A Hyman, and Stephan W Grill. Polarization of par proteins by advective triggering of a pattern-forming system. *Science*, 334(6059):1137–1141, 2011.

[3] Peter Gross, K Vijay Kumar, Nathan W Goehring, Justin S Bois, Carsten Hoege, Frank Jülicher, and Stephan W Grill. Guiding self-organized pattern formation in cell polarity establishment. *Nature physics*, 15(3):293–300, 2019.

[4] William R Holmes, May Anne Mata, and Leah Edelstein-Keshet. Local perturbation analysis: a computational tool for biophysical reaction-diffusion models. *Biophysical journal*, 108(2):230–236, 2015.

[5] Willem H Hundsdorfer, Jan G Verwer, and WH Hundsdorfer. *Numerical solution of time-dependent advection-diffusion-reaction equations*, volume 33. Springer, 2003.

[6] Charles F Lang and Edwin M Munro. Oligomerization of peripheral membrane proteins provides tunable control of cell surface polarity. *Biophysical Journal*, 121(23):4543–4559, 2022.
